# Zeolite A powder and beads from sugarcane bagasse fly ash modified with iron(III) oxide-hydroxide for lead adsorption

**DOI:** 10.1038/s41598-023-29055-4

**Published:** 2023-02-01

**Authors:** Pornsawai Praipipat, Pimploy Ngamsurach, Naritsara Roopkhan

**Affiliations:** 1 Department of Environmental Science, Khon Kaen University, Khon Kaen, 40002 Thailand; 2 Environmental Applications of Recycled and Natural Materials Laboratory (EARN), Khon Kaen University, Khon Kaen, 40002 Thailand

**Keywords:** Engineering, Materials science

## Abstract

The discharging of lead-contaminated wastewater is a concern because of its toxicity to living organisms and water quality resulting in dangerous water consumption, so it is highly recommended to remove lead from wastewater to be below water quality standards for a safe environment. Zeolite A sugarcane bagasse fly ash powder (ZB), zeolite A sugarcane bagasse fly ash powder mixed iron(III) oxide-hydroxide (ZBF), zeolite A sugarcane bagasse fly ash beads (ZBB), zeolite A sugarcane bagasse fly ash powder mixed iron(III) oxide-hydroxide beads (ZBFB), and zeolite A sugarcane bagasse fly ash beads coated iron(III) oxide-hydroxide (ZBBF) were synthesized and characterized in various techniques. Their lead removal efficiencies were investigated by batch experiments, adsorption isotherms, and kinetics. The specific surface area, pore volume, and pore size of ZB were close values to zeolite A standard (STD), and ZBF had the highest specific surface area and the smallest pore size than others. ZB and ZBF demonstrated crystalline phases whereas ZBB, ZBFB, and ZBBF were amorphous phases. The surface morphology of ZB was a cubic shape similar to STD. ZBF demonstrated an agglomerated formation of ZB and iron(III) oxide-hydroxide whereas ZBFB and ZBBF had sphere shapes with coarse surfaces. Si, Al, O, Fe, Na, Ca, O–H, (Si, Al)–O, H_2_O, and D_4_R were detected in all materials. The surface charges of all zeolite A materials had negatively charged at all pH values, and their surfaces increased more negatively charged with increasing pH value which pH 5 illustrated as the highest negatively charged in all materials. Their lead removal efficiencies were higher than 82%. Langmuir isotherm and pseudo-second-order kinetic models were well explained for their adsorption patterns and mechanisms. Finally, ZBBF is a good offer for applying in industrial wastewater treatment systems because of its easy operation and saving costs than ZBF.

## Introduction

Lead contamination in wastewater causes a serious environmental problem to water quality, aquatic organisms, and the environment because of its toxicity, bioaccumulation, and non-degradation. Lead can accumulate through a food chain and causes many dysfunctional systems of nervous, reproductive, blood, respiration, and tissue in humans from human consumption and water use^1,2^. The sources of lead contamination in the environment may be from many industries of battery, paint, steel, and plastic, so their effluents may have lead contamination that needed to remediate before discharging into the environment. Therefore, the lead-contaminated wastewater requires to treat below 0.02 mg/L following USEPA standards for a safe ecosystem in terms of environmental remediation.

Many conventional methods have been used to eliminate heavy metals in wastewater such as coagulation-flocculation, chemical precipitation, ion exchange, oxidation–reduction, and reverse osmosis; however, they have disadvantages of high-cost operation, highly required energy, creating a lot of sludge, and complicated operations^3^. While an adsorption method is a good method with high efficiency, reasonable cost, easy operation, low waste sludge, and many available choices of adsorbents^4^, so this method is a suitable choice for remediating heavy metal in wastewater. Various adsorbents of commercial, natural, wastes of agriculture, industrial, and food have been used for removing heavy metals contaminated from wastewater such as activated carbon, zeolite, chitosan, walnut shells, bagasse, eggshells, banana peels, lemon peels, potato peels, coal fly ash, sawdust, and bagasse fly ash. Especially, wastes from agriculture, industry, and food have been popularly used for eliminating both cationic and anionic heavy metals. For agriculture wastes, corncob, rice husk, peanut shell, coconut shell, and sugarcane bagasse were used for removing lead (Pb), nickel (Ni), copper (Cu), chromium (Cr), and arsenic (As) in wastewater^5–10^. The peels of potato, banana, and lemon are used as food waste adsorbents for lead removal^11–13^. For industrial wastes, sawdust, coal fly ash, and sugarcane bagasse fly ash have been used for removing lead and arsenic in an aqueous solution^14–17^. Therefore, adsorbents from waste are an interesting choice because not only they help to improve water quality for environmental remediation purposes but also using them is another benefit to waste treatment and management in terms of recycling natural resources. Especially, industrial wastes with a big load of waste, if they can be used for another benefit, their waste management can be highly reduced as well.

Sugarcane bagasse fly ash is a waste from sugar factories that has the main chemical elements of silicon (Si), aluminum (Al), iron (Fe), calcium (Ca), magnesium (Mg), potassium (K), and trace elements^18^. The high Si and Al contents in sugarcane bagasse fly ash can be used to synthesize zeolite-type adsorbents for removing heavy metals from wastewater. Especially, zeolite type A gives high heavy metal removals because of its higher surface area with small pore size and high adsorption capacity than other types, so it promotes good adsorption of heavy metals^19^. In previous studies, the synthesized zeolite P from bagasse fly ash is used for eliminating lead and cadmium in an aqueous solution^20^, and the removal of copper is reported by the study of Oliveira et al. by the synthesized zeolite Na-A from sugarcane bagasse fly ash^21^. In addition, the synthesized zeolite A from bagasse fly ash and sugarcane waste ash have been used for removing lead and cadmium in wastewater^19,22^. Although zeolites can adsorb with various toxic metals, improving zeolites to increase adsorption capacity with specific target contaminates is continuously interested. The natural zeolite modified with amine is used for remediating hexavalent chromium (Cr (VI)) in an aqueous media, and MEL-type zeolite nanosheet has been applied as a porous material for water purification from landfill leachate comprising PbCl_2_ and CuCl_2_^23,24^. Moreover, many previous studies have been used various metal oxides of copper oxide (CuO), manganese oxide (MnO_2_), titanium dioxide (TiO_2_), iron (II or III) oxide (Fe_2_O_3_ or Fe_3_O_4_), and iron-zirconium (ZrFe_2_). For the removals of cadmium (Cd^2+^), arsenic (As^5+^), chromium (Cr^2+^), iron (Fe^3+^), and manganese (Mn^2+^) ions in wastewater, zeolite Na-X modified with Fe_3_O_4_, zeolite W modified with iron-zirconium, natural zeolite modified with Fe_3_O_4_, and zeolite 4A modified with titanium dioxide have been applied^25–29^. For the removal of lead (Pb^2+^) ion in an aqueous solution, many studies have used commercial zeolite modified with CuO and Fe_3_O_4_, natural zeolite modified with MnO_2_ and iron oxide, and zeolite Na-X modified with Fe_3_O_4_^25,30–32^. However, no one added iron(III) oxide-hydroxide along with changing material form to improve zeolite A adsorbent for eliminating lead contamination in an aqueous solution. Thus, this study attempted to achieve this purpose by synthesizing novel zeolite A materials from sugarcane bagasse fly ash and investigating their lead removal efficiencies to offer whether adding metal oxide or changing material form help to increase material efficiency. These new zeolite A adsorbents might be a guideline for applying in industries for increasing lead removal efficiency in wastewater in the future as a low-cost adsorbent from industrial waste to achieve both remediating environmental contaminant and reducing waste management.

This study aimed to synthesize five zeolite A adsorbent materials which were zeolite A sugarcane bagasse fly ash powder (ZB), zeolite A sugarcane bagasse fly ash powder mixed iron(III) oxide-hydroxide (ZBF), zeolite A sugarcane bagasse fly ash beads (ZBB), zeolite A sugarcane bagasse fly ash powder mixed iron(III) oxide-hydroxide beads (ZBFB), and zeolite A sugarcane bagasse fly ash beads coated iron(III) oxide-hydroxide (ZBBF) from sugarcane bagasse fly ash for removing lead-contaminated in an aqueous solution. Various characterized techniques of Brunauer–Emmett–Teller (BET), X-ray diffractometer (XRD), Field emission scanning electron microscopy, and focus ion beam (FESEM-FIB) with Energy dispersive X-ray spectrometer (EDX), Fourier transform infrared spectroscopy (FT-IR), and Zetasizer Nano were used to investigate their specific surface area, pore volumes, pore sizes, crystalline structures, surface morphologies, chemical compositions, chemical functional groups, and surface charges. Their lead removal efficiencies were studied through batch experiments, and their adsorption patterns and mechanisms were also determined by linear and nonlinear adsorption isotherms and kinetics.

## Results and discussions

### The physical characteristics

**Figure 1:**
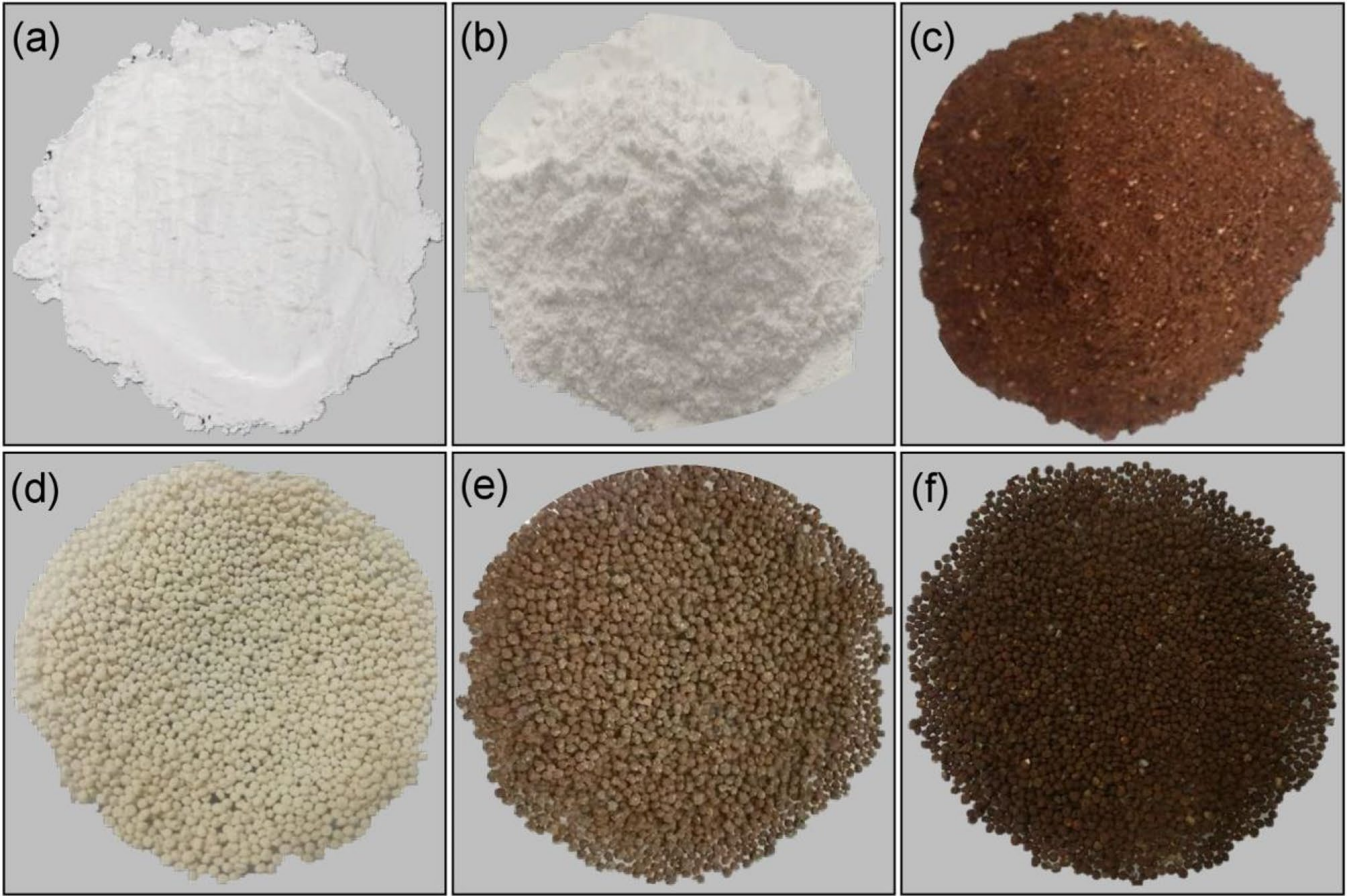
The physical characteristics of (**a**) zeolite A standard (STD), (**b**) zeolite A sugarcane bagasse fly ash powder (ZB), (**c**) zeolite A sugarcane bagasse fly ash powder mixed iron(III) oxide-hydroxide (ZBF), (**d**) zeolite A sugarcane bagasse fly ash beads (ZBB), (**e**) zeolite A sugarcane bagasse fly ash powder mixed iron(III) oxide-hydroxide beads (ZBFB), and (**f**) zeolite A sugarcane bagasse fly ash beads coated iron(III) oxide-hydroxide (ZBBF).

The physical characteristics of zeolite A standard (STD), zeolite A sugarcane bagasse fly ash powder (ZB), zeolite A sugarcane bagasse fly ash powder mixed iron(III) oxide-hydroxide (ZBF), zeolite A sugarcane bagasse fly ash beads (ZBB), zeolite A sugarcane bagasse fly ash powder mixed iron(III) oxide-hydroxide beads (ZBFB), and zeolite A sugarcane bagasse fly ash beads coated iron(III) oxide-hydroxide (ZBBF) are illustrated in Fig. 1a–f. ZB was a white color powder similar to STD demonstrated in Fig. 1a,b. For ZBF, it was an iron-rust color powder correlated to a color of iron(III) oxide-hydroxide added into ZB shown in Fig. 1c. For bead materials, they had sphere shapes with different colors. ZBB was a white beaded color matching the color of ZB presented in Fig. 1d. For ZBFB and ZBBF, they were a light-brown beaded color and a dark-brown beaded color, so the adding iron(III) oxide-hydroxide method might affect a material color shown in Fig. 1e,f, respectively.

### Brunauer–Emmett–Teller (BET) analysis

The specific surface area, pore volume, and pore size of zeolite A standard (STD), zeolite A sugarcane bagasse fly ash powder (ZB), zeolite A sugarcane bagasse fly ash powder mixed iron(III) oxide-hydroxide (ZBF), zeolite A sugarcane bagasse fly ash beads (ZBB), zeolite A sugarcane bagasse fly ash powder mixed iron(III) oxide-hydroxide beads (ZBFB), and zeolite A sugarcane bagasse fly ash beads coated iron(III) oxide-hydroxide (ZBBF) by BET analysis are demonstrated in Table 1. For ZB, it had the specific surface area, pore volume, and pore size are close values to STD which could confirm the ability to synthesize zeolite A from sugarcane bagasse fly ash in this study. For ZBF, its specific surface area and pore volume were increased to 10 times of ZB whereas its pore size was decreased. Thus, it might be possible that ZBF might adsorb lead higher than ZB because it had higher specific surface area and smaller pore size than ZB corresponded to the specific characteristic of a highly efficient adsorbent^12^. As a result, the addition of iron(III) oxide-hydroxide helped to improve material efficiency by increasing specific surface area and decreasing pore size. For ZBB, its specific surface area and pore volume were decreased while its pore size was increased when it was compared to ZB. Thus, the changing material form affected the decreasing specific surface area and pore volume which might result in the decreasing lead removal efficiency. For ZBFB, its specific surface area and pore volume were decreased by approximately 30% and 60% of ZBF while its pore size was increased by approximately 13% of ZBF. For ZBBF, its specific surface area and pore volume were decreased by approximately 20% and 38% of ZBF while its pore size was increased by approximately 10% of ZBF. As a result, the adding iron(III) oxide-hydroxide method into ZB along with changing material form from powder to bead form affected the physiochemical properties of zeolite materials. The coating method was a smaller effect than the mixing method which meant ZBBF might have a higher lead removal efficiency than ZBFB. Finally, all zeolite A materials were classified to be mesoporous size (2–50 nm) followed by the pore dimension identified by the International Union of Pure and Applied Chemistry (IUPAC)^33^.

**Table 1:** The specific surface area, pore volume, and pore size of zeolite A standard (STD), zeolite A sugarcane bagasse fly ash powder (ZB), zeolite A sugarcane bagasse fly ash powder mixed iron(III) oxide-hydroxide (ZBF), zeolite A sugarcane bagasse fly ash beads (ZBB), zeolite A sugarcane bagasse fly ash powder mixed iron(III) oxide-hydroxide beads (ZBFB), and zeolite A sugarcane bagasse fly ash beads coated iron(III) oxide-hydroxide (ZBBF) by BET analysis.

Materials	Surface area (m^2^/g)^a^	Pore volume (cc/g)^b^	Pore size (nm)^c^
STD	10.543	0.028	4.952
ZB	10.756	0.025	5.035
ZBF	106.154	0.271	4.028
ZBB	7.529	0.023	5.891
ZBFB	74.308	0.108	4.632
ZBBF	84.923	0.168	4.431

^a^MultiPoint BET.

^b^Total pore volume for pore with Radius.

^c^Average pore Radius.

### X-ray diffractometer (XRD) analysis

**Figure 2:**
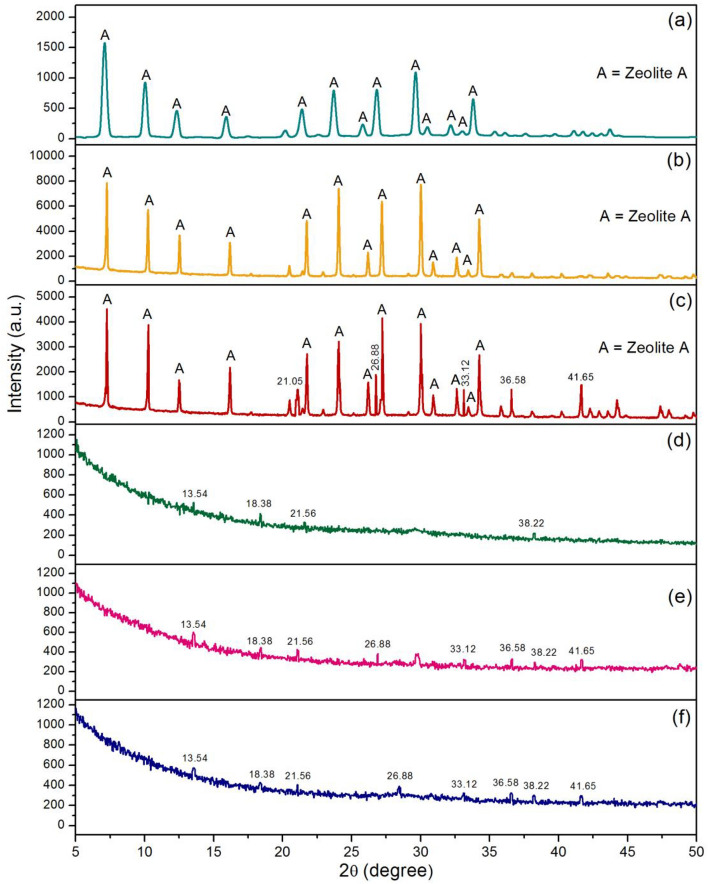
XRD patterns of (**a**) zeolite A standard (STD), (**b**) zeolite A sugarcane bagasse fly ash powder (ZB), (**c**) zeolite A sugarcane bagasse fly ash powder mixed iron(III) oxide-hydroxide (ZBF), (**d**) zeolite A sugarcane bagasse fly ash beads (ZBB), (**e**) zeolite A sugarcane bagasse fly ash powder mixed iron(III) oxide-hydroxide beads (ZBFB), and (**f**) zeolite A sugarcane bagasse fly ash beads coated iron(III) oxide-hydroxide (ZBBF).

The crystalline formations of zeolite A standard (STD), zeolite A sugarcane bagasse fly ash powder (ZB), zeolite A sugarcane bagasse fly ash powder mixed iron(III) oxide-hydroxide (ZBF), zeolite A sugarcane bagasse fly ash beads (ZBB), zeolite A sugarcane bagasse fly ash powder mixed iron(III) oxide-hydroxide beads (ZBFB), and zeolite A sugarcane bagasse fly ash beads coated iron(III) oxide-hydroxide (ZBBF) by XRD analysis are illustrated in Fig. 2a–f. For STD, it was a crystalline phase represented the specific peaks of zeolite A of 7.13°, 10.11°, 12.28°, 16.09°, 21.42°, 24.13°, 26.09°, 27.05°, 30.00°, 30.53°, 31.96°, 32.98° and 33.86° corresponding to JCPDS No. 39-222^34^ shown in Fig. 2a. ZB demonstrated a crystalline phase and found specific peaks of zeolite A similar to STD shown in Fig. 2b, so it could be confirmed the ability of zeolite A synthesis from sugarcane bagasse fly ash. For ZBF, it was a crystalline phase similar to ZB and also found specific peaks of iron(III) oxide-hydroxide of 21.05°, 26.88°, 33.12°, 36.58°, and 41.65° matched to JCPDS No. 29-0713^35^ shown in Fig. 2c which it could confirm the ability to add iron(III) oxide-hydroxide into ZB. For ZBB, it was an amorphous phase and found specific peaks of alginate of 13.54°, 18.38°, 21.56°, and 38.22°^36^ shown in Fig. 2d which could confirm the occurrence of the bead formation in ZBB. For ZBFB, it was an amorphous phase and found both specific peaks of iron(III) oxide-hydroxide and alginate with previously mentioned above, so it could be confirmed the ability of both adding iron(III) oxide-hydroxide and bead formation in ZBFB shown in Fig. 2e. Finally, ZBBF displayed the amorphous phase with detecting both specific peaks of iron(III) oxide-hydroxide and alginate similarly to ZBFB shown in Fig. 2f.

### Field emission scanning electron microscopy and focus ion beam (FESEM-FIB) analysis

**Figure 3:**
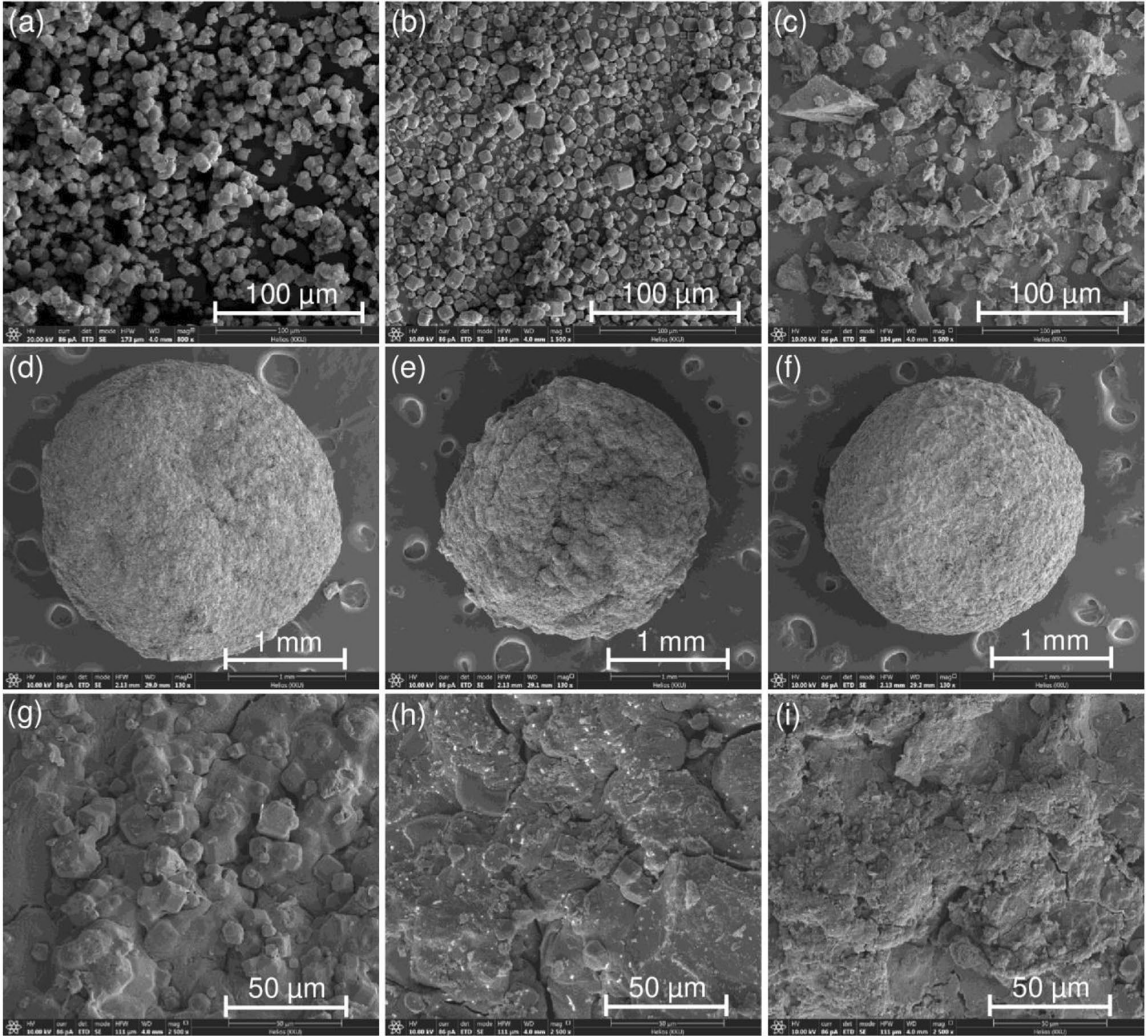
FESEM-FIB micrographs of (**a**) zeolite A standard (STD), (**b**) zeolite A sugarcane bagasse fly ash powder (ZB), (**c**) zeolite A sugarcane bagasse fly ash powder mixed iron(III) oxide-hydroxide (ZBF), (**d**, **g**) zeolite A sugarcane bagasse fly ash beads (ZBB), (**e**, **h**) zeolite A sugarcane bagasse fly ash powder mixed iron(III) oxide-hydroxide beads (ZBFB), and (**f**, **i**) zeolite A sugarcane bagasse fly ash beads coated iron(III) oxide-hydroxide (ZBBF).

FESEM-FIB micrographs of zeolite A standard (STD), zeolite A sugarcane bagasse fly ash powder (ZB), zeolite A sugarcane bagasse fly ash powder mixed iron(III) oxide-hydroxide (ZBF), zeolite A sugarcane bagasse fly ash beads (ZBB), zeolite A sugarcane bagasse fly ash powder mixed iron(III) oxide-hydroxide beads (ZBFB), and zeolite A sugarcane bagasse fly ash beads coated iron(III) oxide-hydroxide (ZBBF) by FESEM-FIB analysis are demonstrated in Fig. 3a–i which the surface morphologies of STD, ZB, and ZBF were investigated by FESEM-FIB at 800 ×, 1500 ×, 1500 × magnification with 100 µm, respectively whereas the surface morphologies of ZBB, ZBFB, and ZBBF were investigated by FESEM-FIB at 130 × magnification with 1 mm and 2500 × magnification with 50 µm. ZB had a cubic shape similar to STD which corresponded to the specific structure shape of zeolite A^19^ shown in Fig. 3a,b, so it could confirm that ZB was zeolite A and corresponded to the BET and XRD results. For ZBF, they have agglomerated formation of ZB and iron(III) oxide-hydroxide which precipitated iron(III) oxide-hydroxide on the surface of ZBF shown in Fig. 3c similarly found in another study^37^. For ZBB, ZBFB, and ZBBF, they had sphere shapes with coarse surfaces at 130 × magnification with 1 mm shown in Fig. 3d–f. In 2500 × magnification with 50 µm, ZBB agglomerated formation and clumped together of ZB and sodium alginate which might be from changing material form presented in Fig. 3g whereas ZBFB and ZBBF were heterogeneous and coarse surfaces shown in Fig. 3h,i.

### Energy dispersive X-ray spectrometer (EDX) analysis

**Figure 4:**
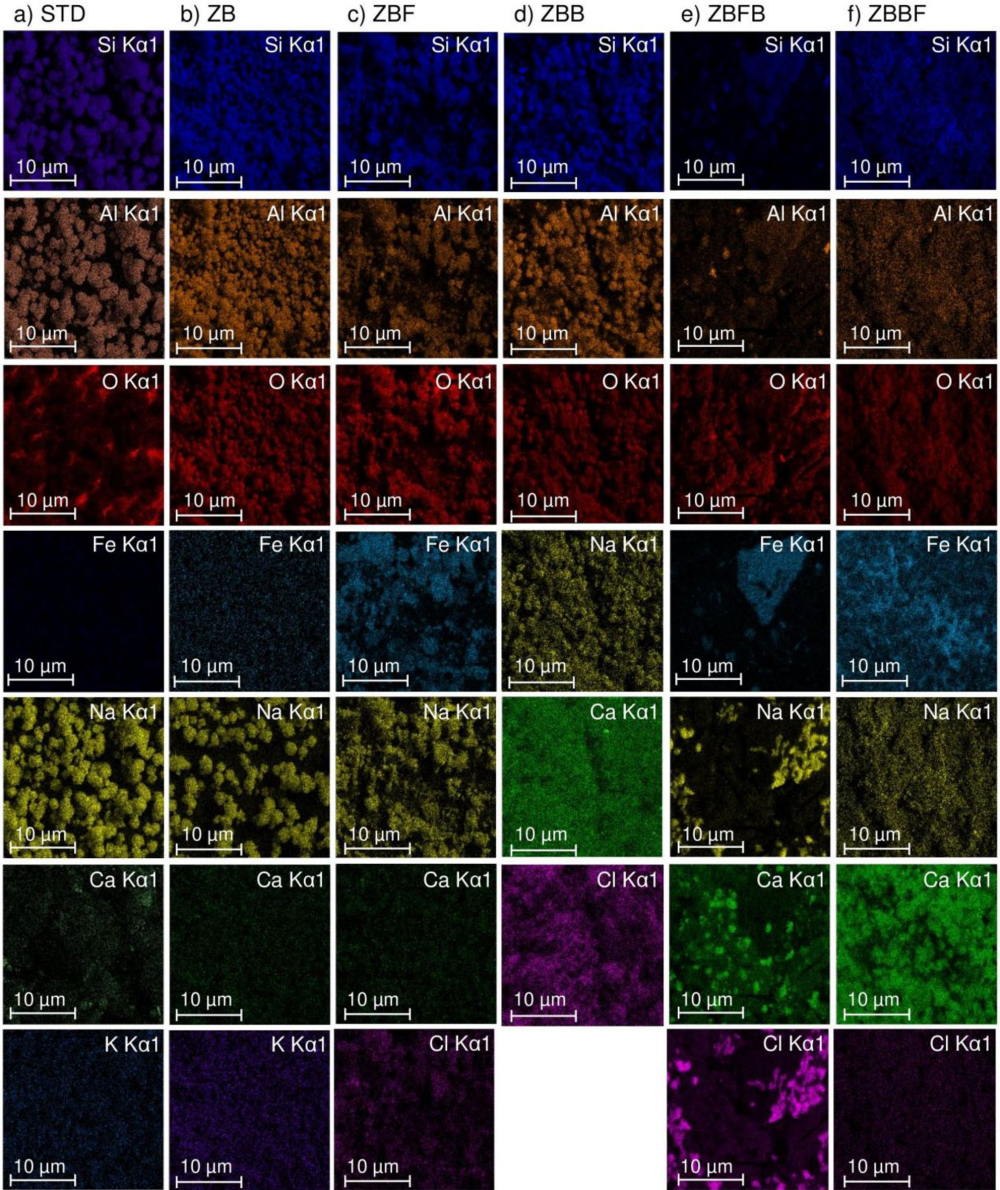
The elemental mapping of (**a**) zeolite A standard (STD), (**b**) zeolite A sugarcane bagasse fly ash powder (ZB), (**c**) zeolite A sugarcane bagasse fly ash powder mixed iron(III) oxide-hydroxide (ZBF), (**d**) zeolite A sugarcane bagasse fly ash beads (ZBB), (**e**) zeolite A sugarcane bagasse fly ash powder mixed iron(III) oxide-hydroxide beads (ZBFB), and (**f**) zeolite A sugarcane bagasse fly ash beads coated iron(III) oxide-hydroxide (ZBBF) on the surface.

The chemical compositions of zeolite A standard (STD), zeolite A sugarcane bagasse fly ash powder (ZB), zeolite A sugarcane bagasse fly ash powder mixed iron(III) oxide-hydroxide (ZBF), zeolite A sugarcane bagasse fly ash beads (ZBB), zeolite A sugarcane bagasse fly ash powder mixed iron(III) oxide-hydroxide beads (ZBFB), and zeolite A sugarcane bagasse fly ash beads coated iron(III) oxide-hydroxide (ZBBF) by EDX analysis are displayed in Table 2, and the dispersions of chemical elements of each zeolite A material on the surface are shown by the elemental mapping in Fig. 4a–f. Six main elements were detected in all materials which were silicon (Si), aluminum (Al), oxygen (O), iron (Fe), sodium (Na), and calcium (Ca). In addition, potassium (K) was only found in STD and ZB whereas chloride was observed in all materials except STD and ZB. Since ZB had main chemical compositions and close percentage by weight similar to STD, it also confirmed ZB was a zeolite A. Especially, the ratio of Si/Al of ZB was close to 1 corresponding to the specific ratio of Si/Al indicating zeolite type A followed the reports of the previous study^19^. For ZBF, it had main chemical elements similar to ZB except K was disappearance. However, the percentages by weight of Si, Al, and O were decreased whereas the percentages by weight of Fe, Na, and Cl were increased because of chemical uses of ferric chloride hexahydrate (FeCl_3_·6H_2_O) and sodium hydroxide (NaOH) in a process of adding iron(III) oxide-hydroxide into ZB. For ZBB, the percentages by weight of Si, Al, O, and Fe were decreased whereas the percentages by weight of Na, Ca, and Cl were increased when compared to ZB because of the chemical uses of sodium alginate and calcium chloride (CaCl_2_) in a bead formation process. Thus, the changing material form affected the increase of Na, Ca, and Cl in ZBB. For ZBFB, the percentages by weight of Si, Al, Na, Ca, and Cl were increased whereas the percentages by weight of O and Fe were decreased when compared to ZBF. For ZBBF, the percentages by weight of Si, Al, O, Na, Ca, and Cl were decreased whereas the percentages by weight of Fe were increased when compared to ZBB. Therefore, the different methods of adding iron(III) oxide-hydroxide affected the percentages by weight of main chemical compositions of zeolite A materials which the results demonstrated that adding iron(III) oxide-hydroxide by coating method could be added iron (Fe) into zeolite A material more than the mixing method.

**Table 2:** The chemical elements of zeolite A standard (STD), zeolite A sugarcane bagasse fly ash powder (ZB), zeolite A sugarcane bagasse fly ash powder mixed iron(III) oxide-hydroxide (ZBF), zeolite A sugarcane bagasse fly ash beads (ZBB), zeolite A sugarcane bagasse fly ash powder mixed iron(III) oxide-hydroxide beads (ZBFB), and zeolite A sugarcane bagasse fly ash beads coated iron(III) oxide-hydroxide (ZBBF) by EDX analysis.

Chemical element (wt%)	STD	ZB	ZBF	ZBB	ZBFB	ZBBF
Si	27.4	26.5	16.2	23.6	18.5	19.8
Al	25.8	24.7	14.9	21.8	16.9	18.3
O	28.6	29.7	27.9	25.8	22.4	18.4
Fe	0.1	0.1	19.8	–	15.4	20.2
Na	17.8	18.5	20.2	23.6	21.5	19.3
Ca	0.1	0.1	0.1	2.9	3.1	2.3
K	0.2	0.4	–	–	–	–
Cl	–	–	0.9	2.3	2.2	1.7

### Fourier transform infrared spectroscopy (FT-IR) analysis

**Figure 5:**
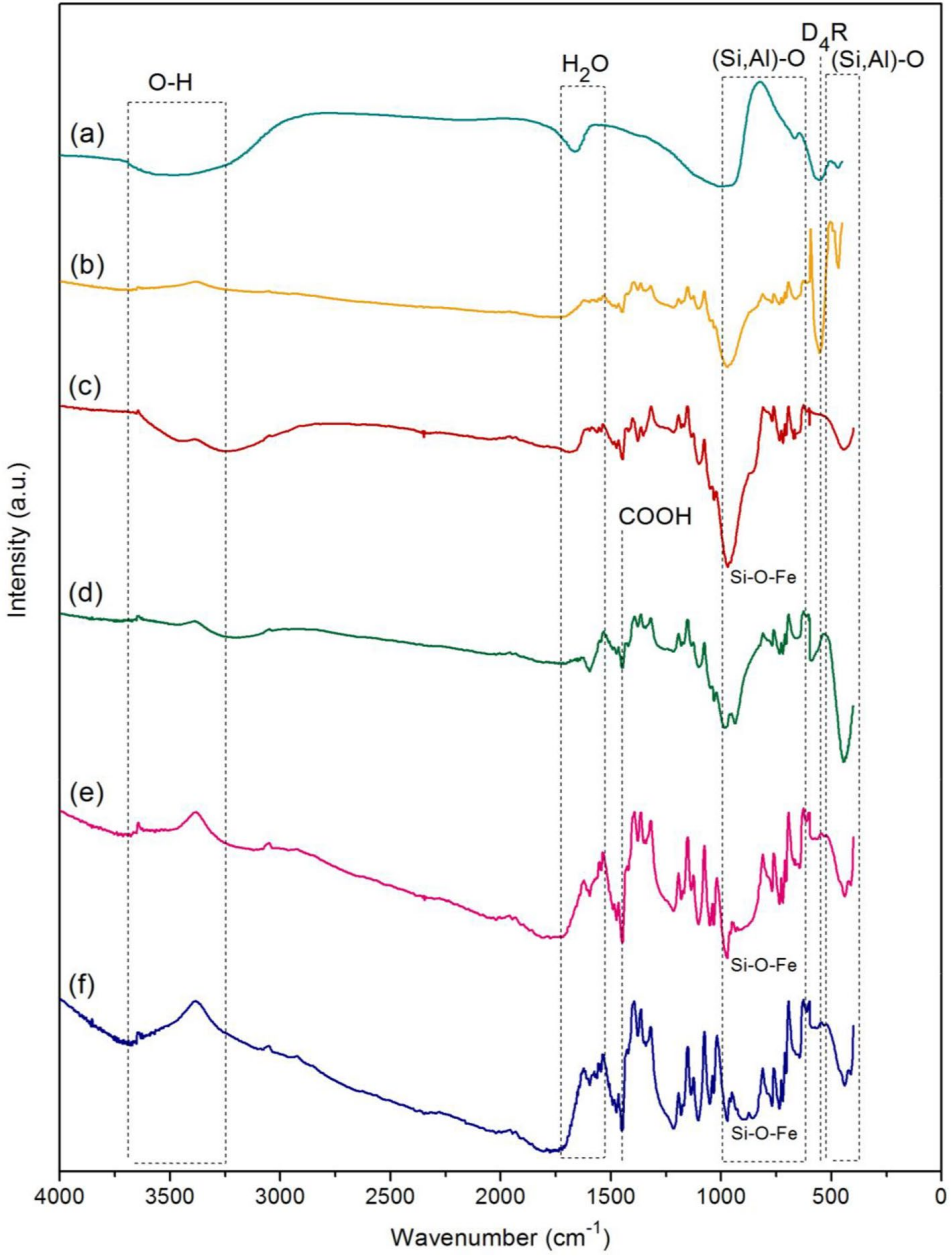
FT-IR spectra of (a) zeolite A standard (STD), (b) zeolite A sugarcane bagasse fly ash powder (ZB), (c) zeolite A sugarcane bagasse fly ash powder mixed iron(III) oxide-hydroxide (ZBF), (d) zeolite A sugarcane bagasse fly ash beads (ZBB), (e) zeolite A sugarcane bagasse fly ash powder mixed iron(III) oxide-hydroxide beads (ZBFB), and (f) zeolite A sugarcane bagasse fly ash beads coated iron(III) oxide-hydroxide (ZBBF).

FT-IR spectra of zeolite A standard (STD), zeolite A sugarcane bagasse fly ash powder (ZB), zeolite A sugarcane bagasse fly ash powder mixed iron(III) oxide-hydroxide (ZBF), zeolite A sugarcane bagasse fly ash beads (ZBB), zeolite A sugarcane bagasse fly ash powder mixed iron(III) oxide-hydroxide beads (ZBFB), and zeolite A sugarcane bagasse fly ash beads coated iron(III) oxide-hydroxide (ZBBF) with the infrared wavelengths of 4000–400 cm^−1^ are shown in Fig. 5a–f. Four main chemical functional groups of O–H, H_2_O, (Si, Al)–O, and D_4_R were detected in all materials. In addition, Si–O–Fe and –COOH were found in materials with adding iron(III) oxide-hydroxide or bead materials. For STD and ZB, they found the same functional groups of O–H of 3480–3450 cm^−1^_,_ (Si, Al)–O asymmetric stretching of 1007–1000 cm^−1^, (Si, Al)–O symmetric stretching of 683–680 cm^−1^, double four-ring (D_4_R) of 577-571 cm^−1^ and Si, Al–O bending of 478–444 cm^−1^ shown in Fig. 5a and b similarly found in another study^19^. D_4_R generally presents the cubic prism formation of zeolite A crystal^38^, so it could be confirmed the presence of zeolite A. Moreover, α-cage, which is the fundamental structure that is linked with D_4_R, confirms the structure of zeolite A^39^. Since the functional groups of ZB were similar to STD, it could be confirmed the ability of zeolite A synthesis by sugarcane bagasse fly ash. For ZBF, ZBB, ZBFB, and ZBBF, they also found the main chemical functional groups similar to STD and ZB; however, they also represented the specific chemical functional groups of adding iron(III) oxide-hydroxide and bead formation. For ZBF, it observed the specific chemical functional groups of Si–O–Fe at 971.31 cm^−1^ which linked to the development of the Si–O–Fe bond into ZB similarly reported by other studies^40,41^ shown in Fig. 5c. For ZBB, it detected the specific chemical functional groups of –COOH symmetric stretching at 1492.47 cm^−1^^42^ shown in Fig. 5d. Finally, ZBFB and ZBBF found the specific chemical functional groups of both Si–O–Fe at 972.98–972.14 cm^−1^ and –COOH symmetric stretching at 1475.50–1474.43 cm^−1^ shown in Fig. 5e and f which were the coordination of alginate with iron(III) oxide-hydroxide similarly reported the carboxyl groups of alginate in an aqueous solution coupled with Fe ion^42^.

### The surface charges of zeolite A materials by zeta potential analysis

**Figure 6:**
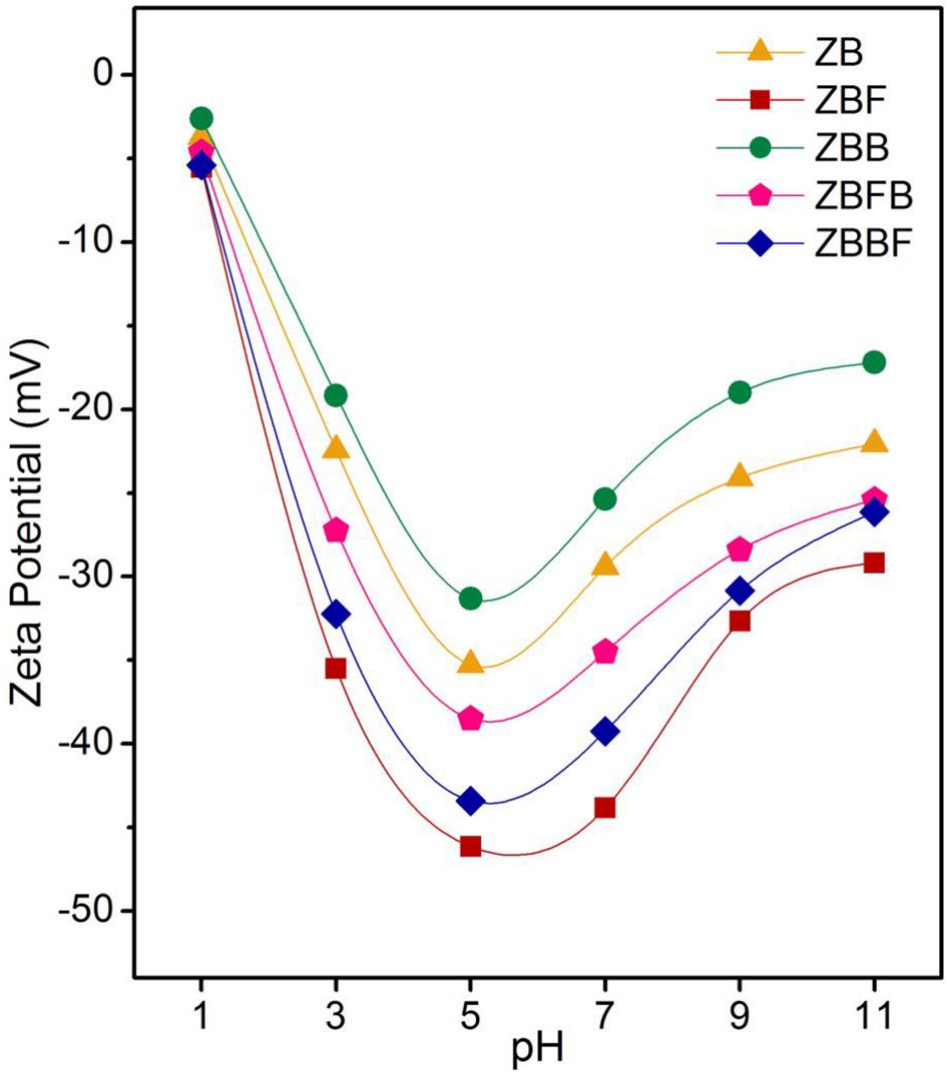
Zeta potentials of zeolite A sugarcane bagasse fly ash powder (ZB), zeolite A sugarcane bagasse fly ash powder mixed iron(III) oxide-hydroxide (ZBF), zeolite A sugarcane bagasse fly ash beads (ZBB), zeolite A sugarcane bagasse fly ash powder mixed iron(III) oxide-hydroxide beads (ZBFB), and zeolite A sugarcane bagasse fly ash beads coated iron(III) oxide-hydroxide (ZBBF) under different pH values.

The zeta potential analysis was used for determining the surface charges of zeolite A sugarcane bagasse fly ash powder (ZB), zeolite A sugarcane bagasse fly ash powder mixed iron(III) oxide-hydroxide (ZBF), zeolite A sugarcane bagasse fly ash beads (ZBB), zeolite A sugarcane bagasse fly ash powder mixed iron(III) oxide-hydroxide beads (ZBFB), and zeolite A sugarcane bagasse fly ash beads coated iron(III) oxide-hydroxide (ZBBF) by a Zetasizer Nano under different pH values of 1, 3, 5, 7, 9, and 11, and the results are illustrated in Fig. 6. The zeta potential values of ZB, ZBF, ZBB, ZBFB, and ZBBF were approximately in ranges of − 3.70 to − 35.27 mV, − 5.53 to − 46.13 mV, − 2.62 to − 31.30 mV, − 4.63 to − 38.50 mV, and − 5.40 to − 43.40 mV, respectively which their surfaces were negative charges, so, they could adsorb lead ions. The increase of pH values from 1 to 5 affected the increase of negative charges on the surfaces of all zeolite A materials because of the highly deprotonating of a proton (H^+^) from hydroxyl groups (OH^−^) of zeolite A materials^19^. After pH 5, their surface charges were more positive charges on the surface which affected the decrease of their lead adsorptions. Especially, zeolite A materials modified with iron(III) oxide-hydroxide in powder and bead forms (ZBF, ZBFB, and ZBBF) had more negative charges on the surface than zeolite A materials without modification (ZB and ZBB), so ZBF, ZBFB, and ZBBF had higher lead adsorption than ZB and ZBB. In Fig. 6, the highest negatively charged of all zeolite A materials were found at pH 5 corresponding to another study that reported the high lead adsorption at pH 5 by zeolite A^19^. Moreover, ZBF demonstrated more negatively charged on the surface than other zeolite A materials in all pH values, so it could adsorb lead ions more than other zeolite A materials.

### Batch adsorption studies

**Figure 7:**
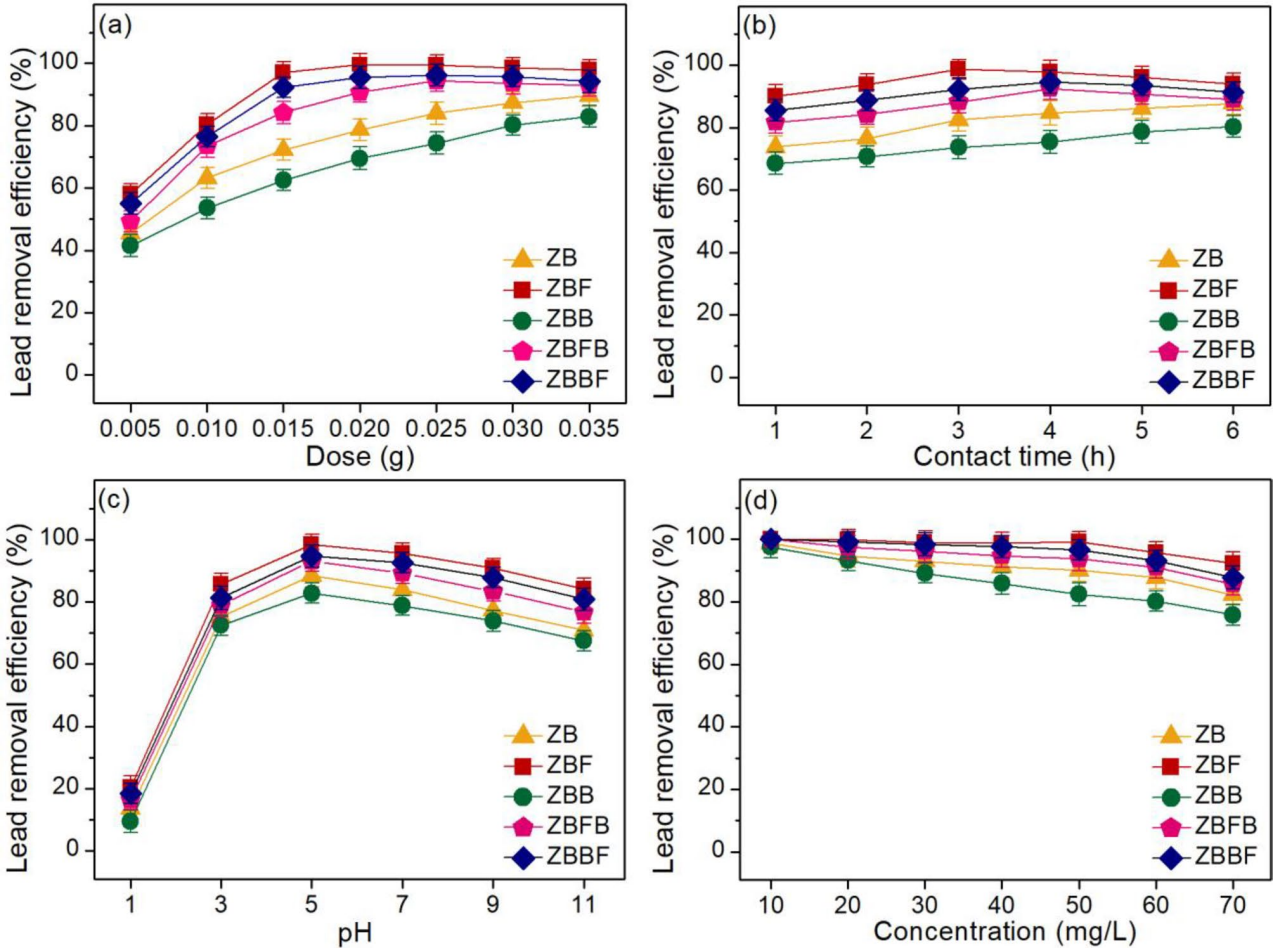
Lead absorption efficiencies of zeolite A sugarcane bagasse fly ash powder (ZB), zeolite A sugarcane bagasse fly ash powder mixed iron(III) oxide-hydroxide (ZBF), zeolite A sugarcane bagasse fly ash beads (ZBB), zeolite A sugarcane bagasse fly ash powder mixed iron(III) oxide-hydroxide beads (ZBFB), and zeolite A sugarcane bagasse fly ash beads coated iron(III) oxide-hydroxide (ZBBF) investigated by (**a**) dosage, (**b**) contact time, (**c**) pH, and (**d**) initial lead concentration.

Lead removal efficiencies of zeolite A sugarcane bagasse fly ash powder (ZB), zeolite A sugarcane bagasse fly ash powder mixed iron(III) oxide-hydroxide (ZBF), zeolite A sugarcane bagasse fly ash beads (ZBB), zeolite A sugarcane bagasse fly ash powder mixed iron(III) oxide- hydroxide beads (ZBFB), and zeolite A sugarcane bagasse fly ash beads coated iron(III) oxide- hydroxide (ZBBF) were investigated by a series of batch experiments with affecting factors of dosage, contact time, pH, and initial lead concentration which their results demonstrated in Fig. 7a–d.

#### The effect of dosage

Figure 7a illustrated the results of does effect of ZB, ZBF, ZBB, ZBFB, and ZBBF on lead adsorptions with the control condition of the lead concentration of 50 mg/L, a sample volume of 200 mL, a contact time of 5 h, pH 5, a temperature of 25 °C, and a shaking speed of 200 rpm. Their lead removal efficiencies were increased with increasing material dose which might be from the increase of active sites of materials. Their highest lead removal efficiencies were 89.69%, 99.60%, 82.93%, 94.41%, and 95.48% at 0.035 g, 0.020 g, 0.035 g, 0.025 g, and 0.020 g, for ZB, ZBF, ZBB, ZBFB, and ZBBF, respectively. Therefore, they were optimum doses of zeolite A materials that were used for studying the contact time effect.

#### The effect of contact time

Figure 7b demonstrated the results of the contact time effect of ZB, ZBF, ZBB, ZBFB, and ZBBF on lead adsorptions with the control condition of the lead concentration of 50 mg/L, a sample volume of 200 mL, pH 5, a temperature of 25 °C, a shaking speed of 200 rpm, and the optimum dose of 0.035 g (ZB) or 0.020 g (ZBF) or 0.035 g (ZBB) or 0.025 g (ZBFB) or 0.020 g (ZBBF). Their lead removal efficiencies were increased with increasing contact time similar to the dose effect. Their highest lead removal efficiencies were 87.72%, 98.65%, 80.35%, 92.50%, and 94.61% at 6 h, 3 h, 6 h, 4 h, and 4 h for ZB, ZBF, ZBB, ZBFB, and ZBBF respectively. Therefore, they were optimum contact times of zeolite A materials which were used for studying the pH effect.

#### The effect of pH

Figure 7c presented the results of the pH effect of ZB, ZBF, ZBB, ZBFB, and ZBBF on lead adsorptions with the control condition of the lead concentration of 50 mg/L, a sample volume of 200 mL, a temperature of 25 °C, a shaking speed of 200 rpm, and the optimum dose of 0.035 g (ZB) or 0.020 g (ZBF) or 0.035 g (ZBB) or 0.025 g (ZBFB) or 0.020 g (ZBBF) and contact time of 6 h (ZB) or 3 h (ZBF) or 6 h (ZBB) or 4 h (ZBFB) or 4 h (ZBBF). Their lead removal efficiencies were increased with increasing pH values from 1 to 5, then they were decreased. Their highest lead removal efficiencies were found at pH 5 with lead removal of 88.45%, 98.55%, 82.84%, 93.20%, and 94.74% for ZB, ZBF, ZBB, ZBFB, and ZBBF, respectively corresponding to the result of zeta potentials of all zeolite A materials that the highest negatively charged was found at pH 5. This result also corresponded to another previous study that reported the highest lead removal efficiency at pH > 4 relating to pH_pzc_ of lead adsorptions in an aqueous solution^19^. Therefore, pH 5 was the optimum pH of all zeolite A materials which were used for studying the concentration effect.

#### The effect of initial lead concentration

Figure 7d examined the results of the initial concentration effect of ZB, ZBF, ZBB, ZBFB, and ZBBF on lead adsorptions with the control condition of the lead concentration of 50 mg/L, a sample volume of 200 mL, a temperature of 25 °C, a shaking speed of 200 rpm, and the optimum dose of 0.035 g (ZB) or 0.020 g (ZBF) or 0.035 g (ZBB) or 0.025 g (ZBFB) or 0.020 g (ZBBF), contact time of 6 h (ZB) or 3 h (ZBF) or 6 h (ZBB) or 4 h (ZBFB) or 4 h (ZBBF), and pH of 5. Their lead removal efficiencies were decreased with increasing concentration because lead ions were more than the available active sites of zeolite A materials similar to the reports by other studies^12^. Lead removal efficiencies from 10 to 70 mg/L of ZB, ZBF, ZBB, ZBFB, and ZBBF were 82.19–98.70%, 92.30–100%, 75.80–97.54%, 85.60–100%, and 87.80–100%, respectively. For the lead concentration of 50 mg/L, their lead removal efficiencies were 90.12%, 99.13%, 82.37%, 93.70%, and 96.53% for ZB, ZBF, ZBB, ZBFB, and ZBBF, respectively, and ZBF demonstrated the highest lead removal efficiency than other materials.

In conclusion, 0.035 g, 6 h, pH 5, 50 mg/L, 0.020 g, 3 h, pH 5, 50 mg/L, 0.035 g, 6 h, pH 5, 50 mg/L, 0.025 g, 4 h, pH 5, 50 mg/L, and 0.020 g, 4 h, pH 5, 50 mg/L were the optimum conditions in dose, contact time, pH, and concentration of ZB, ZBF, ZBB, ZBFB, and ZBBF, respectively, and they could be arranged in order from high to low of ZBF > ZBBF > ZBFB > ZB > ZBB. As a result, the addition of iron(III) oxide-hydroxide into ZB helped to improve material efficiency in both powder and bead forms (ZBF, ZBFB, and ZBBF) whereas only changing material form did not increase lead removal efficiency. As a result, ZBBF might be a good offer for application in industrial wastewater treatment systems with high lead removal efficiency along with easy separation after treatment than other zeolite A materials.

### Adsorption isotherms

**Figure 8:**
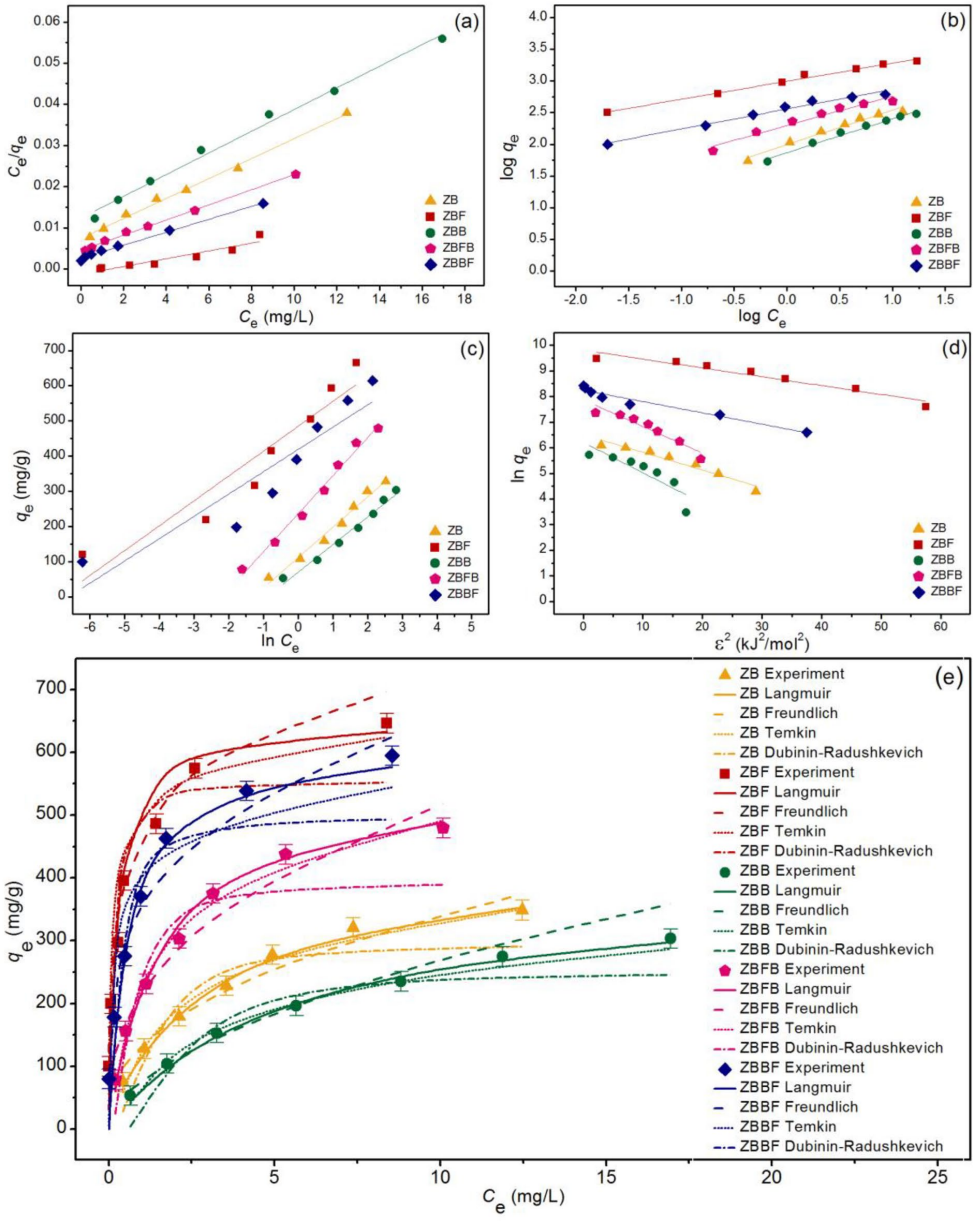
Graphs of (**a**) linear Langmuir, (**b**) linear Freundlich, (**c**) linear Temkin, (**d**) linear Dubinin–Radushkevich, and (**e**) nonlinear adsorption isotherms of zeolite A sugarcane bagasse fly ash powder (ZB), zeolite A sugarcane bagasse fly ash powder mixed iron(III) oxide-hydroxide (ZBF), zeolite A sugarcane bagasse fly ash beads (ZBB), zeolite A sugarcane bagasse fly ash powder mixed iron(III) oxide-hydroxide beads (ZBFB), and zeolite A sugarcane bagasse fly ash beads coated iron(III) oxide-hydroxide (ZBBF) for lead adsorptions.

The adsorption patterns of zeolite A sugarcane bagasse fly ash powder (ZB), zeolite A sugarcane bagasse fly ash powder mixed iron(III) oxide-hydroxide (ZBF), zeolite A sugarcane bagasse fly ash beads (ZBB), zeolite A sugarcane bagasse fly ash powder mixed iron(III) oxide-hydroxide beads (ZBFB), and zeolite A sugarcane bagasse fly ash beads coated iron(III) oxide-hydroxide (ZBBF) on lead adsorptions were determined by linear and nonlinear isotherms of Langmuir, Freundlich, Temkin, and Dubinin–Radushkevich models. Their graphical plotting results are demonstrated in Fig. 8a–e, and their isotherm parameters are illustrated in Table 3.

**Table 3:** The comparison of linear and nonlinear isotherm parameters for lead adsorptions on zeolite A sugarcane bagasse fly ash powder (ZB), zeolite A sugarcane bagasse fly ash powder mixed iron(III) oxide-hydroxide (ZBF), zeolite A sugarcane bagasse fly ash beads (ZBB), zeolite A sugarcane bagasse fly ash powder mixed iron(III) oxide-hydroxide beads (ZBFB), and zeolite A sugarcane bagasse fly ash beads coated iron(III) oxide-hydroxide (ZBBF).

Models	Parameters	ZB	ZBF	ZBB	ZBFB	ZBBF
Linear						
Langmuir	*q*_m_ (mg/g)	416.667	666.667	384.615	555.556	625.000
	*K*_L_ (L/mg)	0.324	3.750	0.210	0.692	2.286
	*R*^2^	0.996	0.992	0.993	0.998	0.995
Freundlich	1*/n*	0.544	0.246	0.533	0.466	0.230
	*K*_F_ (mg/g)(L/mg)^1/n^	98.810	434.911	73.875	197.651	377.920
	*R*^2^	0.974	0.986	0.985	0.961	0.964
Temkin	*b*_T_ (J/mol)	28.778	35.079	31.601	22.937	39.119
	*A*_T_ (L/g)	3.676	721.262	2.459	8.931	746.015
	*R*^2^	0.981	0.901	0.983	0.988	0.858
Dubinin–Radushkevich	*q*_*m*_ (mg/g)	247.324	443.235	222.895	369.186	414.221
	*K*_DR_ (mol^2^/J^2^)	0.182	0.007	0.292	0.006	0.006
	*E* (kJ/mol)	1.656	8.639	1.309	8.909	8.909
	*R*^2^	0.858	0.782	0.829	0.893	0.718
Nonlinear						
Langmuir	*q*_m_ (mg/g)	421.394	633.690	386.699	558.785	632.293
	*K*_L_ (L/mg)	0.304	3.675	0.197	0.687	2.325
	*R*^2^	0.991	0.995	0.994	0.997	0.997
	*R*^2^_adj_	0.990	0.994	0.992	0.996	0.996
	RMSE	8.226	61.219	7.978	13.395	49.306
Freundlich	1*/n*	0.539	0.249	0.541	0.478	0.247
	*K*_F_ (mg/g)(L/mg)^1/n^	104.640	437.571	77.735	204.842	379.780
	*R*^2^	0.894	0.987	0.988	0.965	0.969
	*R*^2^_adj_	0.872	0.984	0.986	0.958	0.963
	RMSE	21.508	25.276	10.839	34.545	36.636
Temkin	*b*_T_ (J/mol)	29.188	39.009	33.324	22.908	43.390
	*A*_T_ (L/g)	3.662	725.365	2.605	8.645	753.162
	*R*^2^	0.981	0.915	0.987	0.989	0.862
	*R*^2^_adj_	0.977	0.898	0.984	0.987	0.834
	RMSE	17.012	81.258	15.275	19.985	90.988
Dubinin–Radushkevich	*q*_*m*_ (mg/g)	278.519	452.419	229.447	372.094	418.132
	*K*_DR_ (mol^2^/J^2^)	0.215	0.008	0.318	0.007	0.007
	*E* (kJ/mol)	1.527	7.772	1.254	8.743	8.745
	*R*^2^	0.677	0.785	0.833	0.894	0.720
	*R*^2^_adj_	0.612	0.742	0.800	0.872	0.664
	RMSE	42.249	84.471	40.235	60.270	76.322

The regression value (*R*^2^) is generally used to investigate which isotherm model well explains the adsorption pattern of zeolite A materials. *R*^2^ values of ZB, ZBF, ZBB, ZBFB, and ZBBF in both linear and nonlinear Langmuir models were higher than Freundlich, Temkin, and Dubinin–Radushkevich models, so their adsorption patterns corresponded to Langmuir isotherm with relating to the physical adsorption. Since the Langmuir model was well explained for the adsorption pattern of all zeolite A materials, Langmuir parameters of *q*_m_ and *K*_L_ values were used to consider which one gave the highest lead adsorption efficiency. The results demonstrated that *q*_m_ and *K*_L_ values of ZBF were higher than others, so it might have the highest lead removal efficiency than other zeolite A materials. Moreover, both linear and nonlinear isotherm models were recommended to plot graphs for confirming the results and protecting against data mistranslation^43–50^.

Finally, the maximum adsorption capacities (*q*_m_) of zeolite A materials in this study were compared with other zeolite adsorbents for lead adsorption reported in Table 4. The *q*_m_ values of all zeolite A materials had higher than previous studies reported in Table 4 except for the studies of Panek et al. and Jangkorn et al^19,51^. As a result, all zeolite A materials were high potential materials for lead adsorption, and can further apply in industrial applications.

**Table 4:** Comparison of the maximum adsorption capacity of various zeolite adsorbents for lead adsorptions.

Adsorbents	Raw materials	*q*_m_ (mg/g)	References
Zeolite	Tuff	16.81	^52^
Zeolite	Laumontite and gismondine	32.80	^53^
Zeolite ZSM-5	–	74.10	^54^
Zeolite K	Coal fly ash	102.00	^55^
Zeolite P	Bagasse fly ash	73.63	^20^
Zeolite P treatment in electrolyte media	Bagasse fly ash	93.20	^20^
Zeolite modified with CuO	Commercial	45.45	^30^
Zeolite modified with Fe_3_O_4_	Commercial	50.00	^30^
Zeolite coated with MnO_2_	Clinoptilolite	40.65	^31^
Zeolite supported nanoscale zero-valent iron	Clinoptilolite	85.90	^56^
Zeolite Na X	Fly ash	575.00	^51^
Zeolite Na X corbon composite	Fly ash	314.00	^51^
3A zeolite (K-LTA)	Venezuelan kaolin	14.64	^57^
Zeolite Na A (hydrothermal)	Rag fly ash	107.60	^58^
Zeolite Na A (fusion)	Rag fly ash	179.70	^58^
Zeolite Na A (fusion-assisted hydrothermal)	Rag fly ash	173.80	^58^
Zeolites A	Coal fly ash	556.00	^19^
Zeolites A	Bagasse fly ash	625.00	^19^
ZB	Sugarcane bagasse fly ash	416.67	This study
ZBF	Sugarcane bagasse fly ash	666.67	This study
ZBB	Sugarcane bagasse fly ash	384.62	This study
ZBFB	Sugarcane bagasse fly ash	555.56	This study
ZBBF	Sugarcane bagasse fly ash	625.00	This study

### Adsorption kinetics

**Figure 9:**
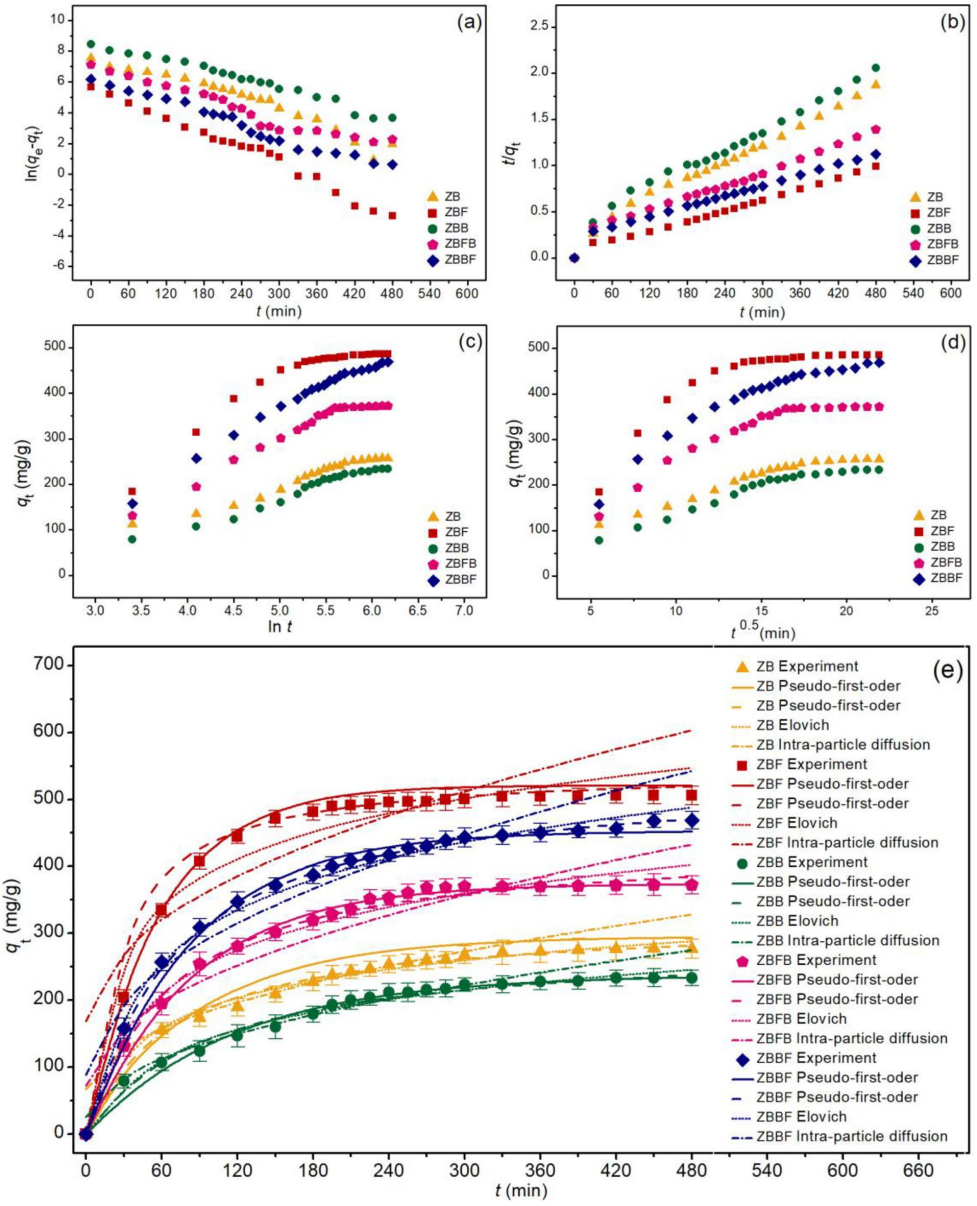
Graphs of (**a**) linear pseudo-first-order, (**b**) linear pseudo-second-order, (**c**) linear elovich model (**d**) linear intra-particle diffusion, and (**e**) nonlinear kinetic models of zeolite A sugarcane bagasse fly ash powder (ZB), zeolite A sugarcane bagasse fly ash powder mixed iron(III) oxide- hydroxide (ZBF), zeolite A sugarcane bagasse fly ash beads (ZBB), zeolite A sugarcane bagasse fly ash powder mixed iron(III) oxide-hydroxide beads (ZBFB), and zeolite A sugarcane bagasse fly ash beads coated iron(III) oxide-hydroxide (ZBBF) for lead adsorptions.

The adsorption mechanisms of zeolite A sugarcane bagasse fly ash powder (ZB), zeolite A sugarcane bagasse fly ash powder mixed iron(III) oxide-hydroxide (ZBF), zeolite A sugarcane bagasse fly ash beads (ZBB), zeolite A sugarcane bagasse fly ash powder mixed iron(III) oxide-hydroxide beads (ZBFB), and zeolite A sugarcane bagasse fly ash beads coated iron(III) oxide-hydroxide (ZBBF) on lead adsorptions were determined by linear and nonlinear isotherms of pseudo-first-order kinetic, pseudo-second-order kinetic, elovich, and intra-particle diffusion models. Their graphical plotting results are demonstrated in Fig. 9a–e, and their isotherm parameters are illustrated in Table 5.

**Table 5:** The comparison of linear and nonlinear kinetic parameters for lead adsorptions on zeolite A sugarcane bagasse fly ash powder (ZB), zeolite A sugarcane bagasse fly ash powder mixed iron(III) oxide-hydroxide (ZBF), zeolite A sugarcane bagasse fly ash beads (ZBB), zeolite A sugarcane bagasse fly ash powder mixed iron(III) oxide-hydroxide beads (ZBFB), and zeolite A sugarcane bagasse fly ash beads coated iron(III) oxide-hydroxide (ZBBF).

Models	Parameters	ZB	ZBF	ZBB	ZBFB	ZBBF
Linear						
Pseudo-first-order kinetic	*q*_e_ (mg/g)	305.363	569.523	289.716	325.187	342.236
	*k*_1_ (min^−1^)	0.013	0.018	0.010	0.011	0.007
	*R*^2^	0.920	0.985	0.978	0.947	0.979
Pseudo-second-order kinetic	*q*_e_ (mg/g)	303.030	526.316	285.714	434.783	500.000
	*k*_2_ (× 10^3^ g/mg min)	0.044	0.074	0.035	0.038	0.041
	*R*^2^	0.994	0.995	0.992	0.996	0.991
Elovich	*α* (mg/g/min)	1.161	1.048	1.252	1.145	1.134
	*β* (g/mg)	0.022	0.011	0.023	0.015	0.012
	*R*^2^	0.951	0.941	0.911	0.943	0.958
Intra-particle diffusion	*k*_i_ (mg/g·min^0.5^)	11.238	19.539	10.888	16.480	20.046
	*C*_i_ (mg/g)	44.227	141.290	25.003	71.780	87.121
	*R*^2^	0.928	0.772	0.946	0.880	0.901
Nonlinear						
Pseudo-first-order kinetic	*q*_e_ (mg/g)	308.111	574.795	297.488	334.004	352.771
	*k*_1_ (min^–1^)	0.011	0.018	0.011	0.012	0.008
	*R*^2^	0.925	0.988	0.982	0.950	0.981
	*R*^2^_adj_	0.921	0.987	0.981	0.948	0.980
	RMSE	12.167	3.311	7.442	7.291	11.111
Pseudo-second-order kinetic	*q*_e_ (mg/g)	313.030	531.060	288.714	441.261	536.208
	*k*_2_ (× 10^3^ g/mg min)	0.049	0.083	0.057	0.046	0.028
	*R*^2^	0.995	0.996	0.993	0.998	0.999
	*R*^2^_adj_	0.994	0.995	0.992	0.997	0.999
	RMSE	8.719	16.217	7.025	25.439	3.409
Elovich	*α* (mg/g/min)	1.265	1.073	1.297	1.172	1.158
	*β* (g/mg)	0.036	0.017	0.026	0.016	0.017
	*R*^2^	0.953	0.942	0.913	0.945	0.962
	*R*^2^_adj_	0.950	0.939	0.909	0.943	0.960
	RMSE	8.007	29.112	8.087	341.869	11.606
Intra-particle diffusion	*k*_i_ (mg/g min^0.5^)	11.938	19.854	11.389	16.565	20.730
	*C*_i_ (mg/g)	46.342	148.351	25.325	73.235	88.530
	*R*^2^	0.930	0.974	0.947	0.883	0.905
	*R*^2^_adj_	0.926	0.972	0.944	0.877	0.900
	RMSE	17.543	59.647	16.939	34.368	37.389

Normally, the regression value (*R*^2^) is used to decide which kinetic model well explains the adsorption rate and mechanism of zeolite A materials. *R*^2^ values of ZB, ZBF, ZBB, ZBFB, and ZBBF in both linear and nonlinear pseudo-second-order kinetic models were higher than pseudo-first-order kinetic, elovich, and intra-particle diffusion models, so their adsorption rate and mechanism of all zeolite A materials corresponded to a pseudo-second-order kinetic model with relating to the chemisorption process with a heterogeneous adsorption. Since a pseudo-second-order kinetic model was the best-fitted model to explain their adsorption mechanisms, its parameters of *q*_e_ and *k*_2_ values were used to consider which one gave the highest lead adsorption efficiency with a fast reaction. The results illustrated *q*_e_ and *k*_2_ values of ZBF were higher than others, so it might have the highest lead removal efficiency with fast reaction than other zeolite A materials. Finally, the graph plotting of both linear and nonlinear kinetic models was also recommended for correct data translations^43–50^.

## The possible mechanisms for lead adsorption by zeolite A materials

**Figure 10:**
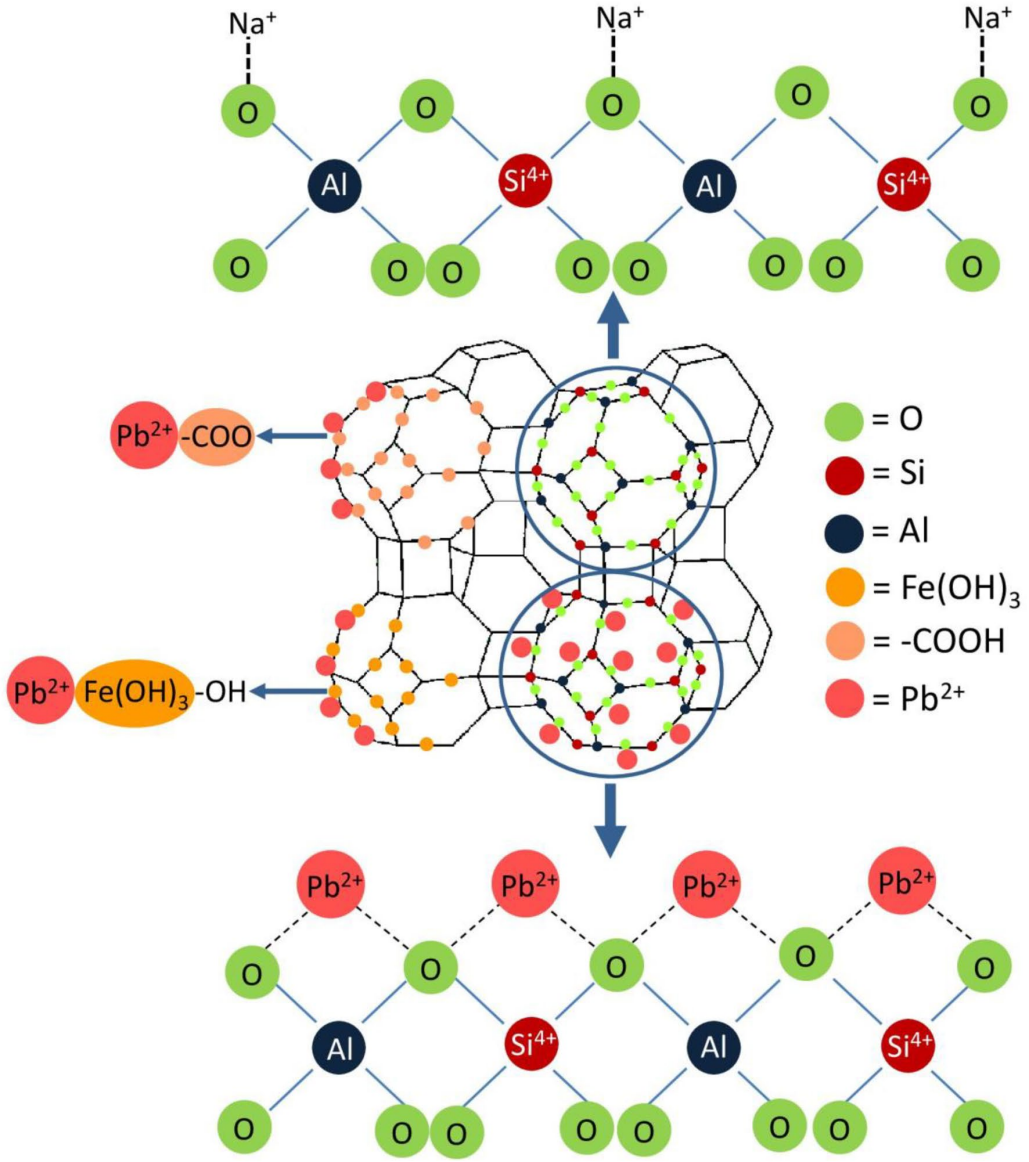
The possible mechanisms for lead adsorption by zeolite A materials.

The possible mechanisms of lead adsorptions on zeolite A materials are demonstrated in Fig. 10 which modified the idea from Fan et al., Jangkorn et al., Rahimi et al., and Isawi^19,59–61^. Firstly, lead ions (Pb^2+^) as positively charged are adsorbed by zeolite A materials by attaching to oxygen ions (O^2−^) in the structure of zeolite A materials as negatively charged following Lewis acid–base theory creating the strong coordinate bond of lead ions (Pb^2+^) with oxygen ions (O^2−^). Secondly, the hydroxyl groups (OH^−^) of zeolite A materials create the complex formation between hydroxyl group (OH^−^) and iron(III) oxide-hydroxide (Fe(OH)_3_) by sharing electron pair and lead ions (Pb^2+^) are adsorbed by this complex molecule. In addition, the carboxylic groups (–COOH) of sodium alginate formed on the surface of zeolite A materials will adsorb lead ion (Pb^2+^) by deprotonating hydrogen (H^+^) of carboxylic groups (–COOH) to be negatively charged. From zeta potential analysis, it demonstrated the negatively charged of all zeolite A materials in all pH values, and pH 5 represented their highest negatively charged in all materials. In addition, the addition of iron(III) oxide-hydroxide in zeolite A materials helped to increase negatively charged on their surfaces increasing lead adsorption, especially ZBF. Finally, lead removal by zeolite A materials might occur through an ion exchange process by releasing hydrogen ions (H^+^) in hydroxyl groups (OH^−^) of zeolite A materials instead of lead ions (Pb^2+^).

## Conclusions

Five zeolite A materials of zeolite A sugarcane bagasse fly ash powder (ZB), zeolite A sugarcane bagasse fly ash powder mixed iron(III) oxide-hydroxide (ZBF), zeolite A sugarcane bagasse fly ash beads (ZBB), zeolite A sugarcane bagasse fly ash powder mixed iron(III) oxide-hydroxide beads (ZBFB), and zeolite A sugarcane bagasse fly ash beads coated iron(III) oxide-hydroxide (ZBBF) were synthesized from sugarcane bagasse fly ash for lead adsorptions in an aqueous solution. The physicochemical properties of ZB were close values to the zeolite A standard (STD), so it could confirm the ability to synthesize zeolite A in this study. In addition, ZBF had the highest specific surface area and smallest pore size than other zeolite A materials, so it might adsorb lead higher than others. ZB and ZBF demonstrated the crystalline phases whereas ZBB, ZBFB, and ZBBF were amorphous phases. Moreover, the specific peaks of iron(III) oxide-hydroxide or sodium alginate were also detected in ZBF, ZBB, ZBFB, and ZBBF. The surface morphology of ZB was a cubic shape similar to STD related to the specific shape of zeolite A. ZBF demonstrated an agglomerated formation of ZB and iron(III) oxide-hydroxide whereas ZBFB and ZBBF had sphere shapes with coarse surfaces. Six main chemical compositions were Si, Al, O, Fe, Na, and Ca were observed in all zeolite A materials. The four main chemical functional groups of all materials were O–H, (Si, Al)–O, H_2_O, and D_4_R whereas –COOH and Si–O–Fe were only found in materials with adding iron(III) oxide-hydroxide or bead material. The surface charges of all zeolite A materials had negatively charged at all pH values, and their surfaces increased more negatively charged with increasing pH value which pH 5 illustrated as the highest negatively charged in all materials. For batch experiments, the optimum conditions of ZB, ZBF, ZBB, ZBFB, and ZBBF were 0.035 g, 6 h, pH 5, 50 mg/L, 0.020 g, 3 h, pH 5, 50 mg/L, 0.035 g, 6 h, pH 5, 50 mg/L, 0.025 g, 4 h, pH 5, 50 mg/L, and 0.020 g, 4 h, pH 5, 50 mg/L, respectively. Lead removal efficiencies of ZB, ZBF, ZBB, ZBFB, and ZBBF at 50 mg/L were 90.12%, 99.13%, 82.37%, 93.70%, and 96.53%, respectively, and it could be arranged in order from high to low of ZBF > ZBBF > ZBFB > ZB > ZBB. Thus, adding iron(III) oxide-hydroxide into ZB improved material efficiency in both powder and bead forms while only changing material form did not increase the lead removal efficiency of zeolite A material. All zeolite A materials corresponded to Langmuir isotherm and pseudo-second-order kinetic models, so their adoption patterns and mechanisms were explained by the physical adsorption and chemisorption with a heterogeneous process, respectively. Although ZBF demonstrated the highest lead removal efficiency than other zeolite A materials, ZBBF might be a good offer for applications in industrial wastewater treatment systems because it was easier separation after treatment than ZBF.

For future works, the desorption experiments need to investigate possibly reusable zeolite A materials, and continuous flow experiments are also necessary to investigate the possible application in the systems of industrial wastewater treatment.

## Materials and methods

### Raw material

Sugarcane bagasse fly ash was obtained by a sugar factory located in Khon Kaen province, Thailand, and then they were sieved in size of 125 µm before use. For a pretreatment, 20 g of sugarcane bagasse fly ash were burned by a furnace (Vulcan, 3-1750, Canada) with increasing temperature in a step up of 20 °C per min until 600 °C for 4 h. Then, they were cooled at room temperature and kept in a desiccator before use for zeolite A synthesis.

### Chemicals

All chemicals used in this study were analytical grades (AR) without purification. Sodium aluminate (NaAlO_2_) (Sigma-Aldrich, Germany) and sodium hydroxide (NaOH) (RCI Labscan, Thailand) were used for the synthesis of zeolite A sugarcane bagasse fly ash powder. To confirm the occurrence of zeolite A, commercial zeolite A (Sigma-Aldrich, Germany) was used as a representative zeolite A standard (STD). Ferric chloride hexahydrate (FeCl_3_·6H_2_O) (RCI Labscan, Thailand) was used for the synthesis of zeolite A sugarcane bagasse fly ash mixed or coated iron(III) oxide-hydroxide in powder and bead materials. Sodium alginate (NaC_6_H_7_O_6_) (Merck, Germany) and calcium chloride (CaCl_2_) (Kemaus, New Zealand) were used for the bead formations. Lead nitrate (Pb(NO_3_)_2_) (QRëC, New Zealand) was used for the synthetic wastewater preparation. For pH adjustments, 1% sodium hydroxide (NaOH) (RCI Labscan, Thailand) and 1% nitric acid (HNO_3_) (Merck, Germany) were used.

### Synthesis of zeolite A sugarcane bagasse fly ash materials

**Figure 11:**
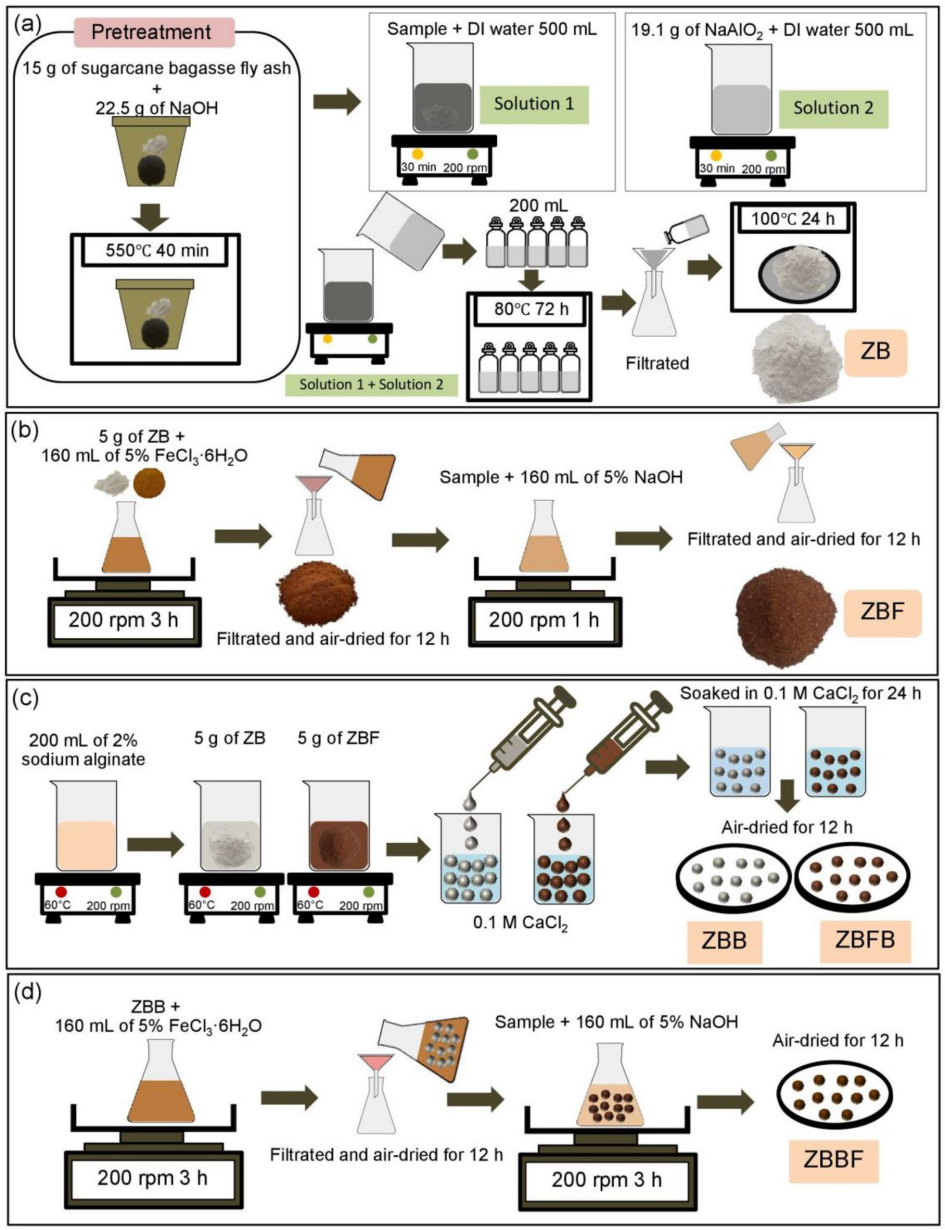
A flow diagram of the synthesis methods of (**a**) zeolite A sugarcane bagasse fly ash powder (ZB), (**b**) zeolite A sugarcane bagasse fly ash powder mixed iron(III) oxide-hydroxide (ZBF), (**c**) zeolite A sugarcane bagasse fly ash beads (ZBB) or zeolite A sugarcane bagasse fly ash powder mixed iron(III) oxide-hydroxide beads (ZBFB), and (**d**) zeolite A sugarcane bagasse fly ash beads coated iron(III) oxide-hydroxide (ZBBF).

The synthesis of five zeolite A sugarcane bagasse fly ash materials which were zeolite A sugarcane bagasse fly ash powder (ZB), zeolite A sugarcane bagasse fly ash powder mixed iron(III) oxide-hydroxide (ZBF), zeolite A sugarcane bagasse fly ash beads (ZBB), and zeolite A sugarcane bagasse fly ash powder mixed iron(III) oxide-hydroxide beads (ZBFB), and zeolite A sugarcane bagasse fly ash beads coated iron(III) oxide-hydroxide (ZBBF) demonstrated in Fig. 11a–d and their synthesis details were below:

#### Zeolite A sugarcane bagasse fly ash powder (ZB)

Zeolite A bagasse fly ash (ZB) was synthesized by a two-stage method followed by a method of Jangkorn et al^19^. Firstly, 15 g of pre-treatment sugarcane bagasse fly ash and 22.5 g of NaOH were added to a nickel crucible (United scientific, NCR100, USA) and heated by a furnace (Carbolite, CWF, England) of 550 °C for 40 min. After that, the sample was added to a 1000 mL beaker containing 500 mL of deionized water (DI water), and then it was mixed by a magnetic stirrer of 200 rpm for 30 min called solution 1. Next, 19.1 g of NaAlO_2_ were added to a 1000 mL beaker containing 500 mL of DI water, and it was mixed by a magnetic stirrer of 200 rpm for 30 min called solution 2. Then, solution 2 was slowly added to a 1000 mL beaker containing solution 1, and they were homogeneously mixed by a magnetic stirrer of 200 rpm for 30 min. Next, 1000 mL of sample were separated to be 200 mL to add in 250 mL of polyethylene bottle in 5 bottles. Then, they were heated by a hot air oven (Binder, FED53, Germany) of 80 °C for 72 h, filtrated, and rinsed with DI water. Finally, they were dried in a hot air oven (Binder, FED53, Germany) of 100 °C for 24 h and kept in a desiccator before use called zeolite A sugarcane bagasse fly ash powder (ZB).

#### Zeolite A sugarcane bagasse fly ash powder mixed iron(III) oxide-hydroxide (ZBF)

5 g of ZB were added to 500 mL of Erlenmeyer flask containing 160 mL of 5% FeCl_3_·6H_2_O, and they were mixed by an orbital shaker (GFL, 3020, Germany) of 200 rpm for 3 h. Then, they were filtrated and air-dried at room temperature for 12 h. After that, they were added to 500 mL of Erlenmeyer flask containing 160 mL of 5% NaOH, and they were mixed by an orbital shaker (GFL, 3020, Germany) of 200 rpm for 1 h. Then, they were filtered, air-dried at room temperature for 12 h, and kept in a desiccator before use called zeolite A sugarcane bagasse fly ash powder mixed iron(III) oxide-hydroxide (ZBF)*.*

#### Zeolite A sugarcane bagasse fly ash beads (ZBB) or zeolite A sugarcane bagasse fly ash powder mixed iron(III) oxide-hydroxide beads (ZBFB)

5 g of ZB or ZBF were added to 500 mL of a beaker containing 200 mL of 2% NaC_6_H_7_O_6_, and then they were homogeneously mixed and heated by a hot plate (Ingenieurbüro CAT, M. Zipperer GmbH, M 6, Germany) at 60 °C with a constant stirring of 200 rpm. Then, they were dropped by drop by using a 10 mL syringe with a needle size of 1.2 × 40 mm into 250 mL of 0.1 M CaCl_2_. The beaded samples were soaked in 0.1 M CaCl_2_ for 24 h, and then they were filtered and rinsed with DI water. After that, they were air-dried at room temperature for 12 h and kept in a desiccator before use called zeolite A sugarcane bagasse fly ash beads (ZBB) or zeolite A sugarcane bagasse fly ash powder mixed iron(III) oxide-hydroxide beads (ZBFB).

#### Zeolite A sugarcane bagasse fly ash beads coated iron(III) oxide-hydroxide (ZBBF)

Firstly, ZBB were added to 500 mL of Erlenmeyer flask containing 160 mL of 5% FeCl_3_·6H_2_O, and they were mixed by an orbital shaker (GFL, 3020, Germany) of 200 rpm for 3 h. Then, they were filtrated and air-dried at room temperature for 12 h. After that, they were added to 500 mL of Erlenmeyer flask containing 160 mL of 5% NaOH, and they were mixed by an orbital shaker (GFL, 3020, Germany) of 200 rpm for 1 h. Then, they were filtered, air-dried at room temperature for 12 h, and kept in a desiccator before use called zeolite A sugarcane bagasse fly ash beads coated iron(III) oxide-hydroxide (ZBBF).

### Characterizations of zeolite A materials

Several characterized techniques were used to investigate the specific surface area, pore volumes, pore sizes, crystalline structures, surface morphologies, chemical compositions, chemical functional groups, and surface charges of zeolite A sugarcane bagasse fly ash powder (ZB), zeolite A sugarcane bagasse fly ash powder mixed iron(III) oxide-hydroxide (ZBF), zeolite A sugarcane bagasse fly ash beads (ZBB), zeolite A sugarcane bagasse fly ash powder mixed iron(III) oxide-hydroxide beads (ZBFB), and zeolite A sugarcane bagasse fly ash beads coated iron(III) oxide-hydroxide (ZBBF) by using Brunauer–Emmett–Teller (BET) (Quantachrome, QUADRASORB evo™, Austria) by isothermal nitrogen gas (N_2_) adsorption–desorption at 77.3 K and degas temperature of 80 °C for 6 h, X-ray diffractometer (XRD) (PANalytical, EMPYREAN, United Kingdom) in the range of 2θ = 5–50°, Field emission scanning electron microscopy and focus ion beam (FESEM-FIB) with Energy dispersive X-ray spectrometer (EDX) (FEI, Helios NanoLab G3 CX, USA), Fourier transform infrared spectroscopy (FT-IR) (Thermo Fisher Scientific, Nicolet 6700, USA), and Zetasizer Nano (Malvern, Zetasizer Nano ZS, United Kingdom), respectively.

### Batch adsorption studies

A series of batch adsorption studies were carried out for investigating lead adsorption efficiencies on zeolite A sugarcane bagasse fly ash powder (ZB), zeolite A sugarcane bagasse fly ash powder mixed iron(III) oxide-hydroxide (ZBF), zeolite A sugarcane bagasse fly ash beads (ZBB), zeolite A sugarcane bagasse fly ash powder mixed iron(III) oxide-hydroxide beads (ZBFB), and zeolite A sugarcane bagasse fly ash beads coated iron(III) oxide-hydroxide (ZBBF) with studying affecting factors of dose, contact time, pH, and concentration, and lead concentrations were analyzed by an Atomic Adsorption Spectrophotometer (AAS) (PerkinElmer, PinAAcle 900F, USA). The details of batch adsorption studies were described below:

#### Effect of dose

The dosages of zeolite A materials from 0.005 to 0.035 g with the control condition of the initial lead concentration of 50 mg/L, a sample volume of 200 mL, a shaking speed of 200 rpm, a contact time of 5 h, pH 5, and a temperature of 25 °C were used for studying dose effect for lead adsorptions on ZB, ZBF, ZBB, ZBFB, and ZBBF. The lowest dose which represented the highest lead removal efficiency was used as the optimum dose for studying the effect of contact time.

#### Effect of contact time

The contact times from 1 to 5 h with the control condition of the initial lead concentration of 50 mg/L, a sample volume of 200 mL, a shaking speed of 200 rpm, pH 5, a temperature of 25 °C, and the optimum dose were used for investigating contact time effect for lead adsorptions on ZB, ZBF, ZBB, ZBFB, and ZBBF. The lowest contact time which represented the highest lead removal efficiency was chosen as the optimum contact time for studying of pH effect.

#### Effect of pH

The pH values of 1, 3, 5, 7, 9, and 11 with the control condition of initial lead concentration of 50 mg/L, a sample volume of 200 mL, a shaking speed of 200 rpm, a temperature of 25 °C, and the optimum dose and contact time were used to investigate the effect of pH for lead adsorptions on ZB, ZBF, ZBB, ZBFB, and ZBBF. The pH value showed the highest lead removal efficiency which was used as the optimum pH for studying the effect of concentration.

#### Effect of concentration

Lead concentrations from 10 to 70 mg/L with the control condition of a sample volume of 200 mL, a shaking speed of 200 rpm, a temperature of 25 °C, and the optimum dose, contact time, and pH were used to study the effect of concentration for lead adsorptions on ZB, ZBF, ZBB, ZBFB, and ZBBF.

Lead removal efficiency of zeolite A materials in the percentage were calculated by Eq. (1)

1 $${\text{Lead}}\;{\text{ removal}}\;{\text{efficiency}}\left( \% \right) = \, (C_{0} - C_{{\text{e}}} )/C_{0} \times {1}00$$

where *C*_e_ is the equilibrium of lead concentration in the solution (mg/L), *C*_0_ is the initial lead concentration (mg/L).

### Adsorption isotherms

Linear and nonlinear Langmuir, Freundlich, Temkin, and Dubinin–Radushkevich models are used to investigate the adsorption patterns of zeolite A sugarcane bagasse fly ash powder (ZB), zeolite A sugarcane bagasse fly ash powder mixed iron(III) oxide-hydroxide (ZBF), zeolite A sugarcane bagasse fly ash beads (ZBB), zeolite A sugarcane bagasse fly ash powder mixed iron(III) oxide-hydroxide beads (ZBFB), and zeolite A sugarcane bagasse fly ash beads coated iron(III) oxide-hydroxide (ZBBF) for lead adsorptions. Graphs of linear Langmuir, Freundlich, Temkin, and Dubinin–Radushkevich isotherms were plotted by *C*_e_*/q*_e_ versus *C*_e,_ log *q*_e_ versus log *C*_e_, *q*_e_ versus ln *C*_e_, and ln *q*_e_ versus *ε*^2^, respectively whereas graphs of their nonlinear were plotted by *q*_e_ versus *C*_e_*.* All linear and nonlinear adsorption models were analyzed by following Eqs. (2)–(9)^62–65^:

#### Langmuir isotherm

2 $${\text{Linear}}: C_{{\text{e}}} /q_{{\text{e}}} = \, 1/q_{{\text{m}}} K_{{\text{L}}} + C_{{\text{e}}} /q_{{\text{m}}}$$

3 $${\text{Nonlinear}}:q_{e} = \, q_{{\text{m}}} K_{{\text{L}}} C_{{\text{e}}} /{1} + K_{{\text{L}}} C_{{\text{e}}}$$

#### Freundlich isotherm

4 $${\text{Linear}}: {\text{log}}q_{{\text{e}}} = {\text{log}}K_{{\text{F}}} + {1}/n \,{\text{log}}C_{{\text{e}}}$$

5 $${\text{Nonlinear}}:q_{{\text{e}}} = \, K_{{\text{F}}} C_{{\text{e}}}^{{{1}/n}}$$

#### Temkin isotherm

6 $${\text{Linear}}: q_{e} = \, RT/b_{{\text{T}}} \, \ {\text{ln}} \; A_{{\text{T}}} + RT/b_{{\text{T}}} \; \ {\text{ln}} \; C{\text{e}}$$

7 $${\text{Nonlinear}}: q_{{\text{e}}} = RT/b_{{\text{T}}} \; {\text{ln}}\; A_{{\text{T}}} C_{{\text{e}}}$$

#### Dubinin–Radushkevich isotherm

8 $${\text{Linear}}: \ln q_{e} = \, \ln q_{m} {-}K_{{{\text{DR}}}} \varepsilon^{2}$$

9 $${\text{Nonlinear}}:q_{{\text{e}}} = q_{{\text{m}}} {\text{exp}}( - K_{{{\text{DR}}}} \varepsilon^{{2}} )$$

where *C*_e_ is the equilibrium of lead concentration (mg/L), *q*_e_ is the amount of adsorbed lead on adsorbent materials (mg/g), *q*_m_ is indicated the maximum amount of lead adsorption on adsorbent materials (mg/g), *K*_*L*_ is the adsorption constant (L/mg). *K*_F_ is the constant of adsorption capacity (mg/g)(L/mg)^1/n^, and 1/*n* is the constant depicting the adsorption intensity. *R* is the universal gas constant (8.314 J/mol K), *T* is the absolute temperature (K), *b*_T_ is the constant related to the heat of adsorption (J/mol), and *A*_*T*_ is the equilibrium binding constant corresponding to the maximum binding energy (L/g). *q*_*m*_ is the theoretical saturation adsorption capacity (mg/g), *K*_DR_ is the activity coefficient related to mean adsorption energy (mol^2^/J^2^), and *ε* is the Polanyi potential (J/mol).

For adsorption isotherm experiments, 0.035 g of ZB, 0.020 g of ZBF, 0.035 g of ZBB, 0.025 g of ZBFB, and 0.020 g of ZBBF were added to 500 mL Erlenmeyer flasks with variable lead concentrations from 10 to 70 mg/L. The control condition of ZB, ZBF, ZBB, ZBFB, and ZBBF was a sample volume of 200 mL, a shaking speed of 200 rpm, pH 5, a temperature of 25 °C, and a contact time of 6 h for ZB, 3 h for ZBF, 6 h for ZBB, 4 h for ZBFB, and 4 h for ZBBF.

### Adsorption kinetics

Linear and nonlinear pseudo-first-order kinetic, pseudo-second-order kinetic, elovich, and intra-particle diffusion models are used to study the adsorption mechanisms of zeolite A sugarcane bagasse fly ash powder (ZB), zeolite A sugarcane bagasse fly ash powder mixed iron(III) oxide-hydroxide (ZBF), zeolite A sugarcane bagasse fly ash beads (ZBB), zeolite A sugarcane bagasse fly ash powder mixed iron(III) oxide-hydroxide beads (ZBFB), and zeolite A sugarcane bagasse fly ash beads coated iron(III) oxide-hydroxide (ZBBF) for lead adsorptions. Graphs of linear pseudo-first-order, pseudo-second-order, elovich, and intra-particle diffusion models were plotted by ln(*q*_e_ − *q*_t_) versus time (*t*), *t*/*q*_t_ versus time (*t*), *q*_t_ versus ln *t*, and *q*_t_ versus time (*t*^0.5^), respectively whereas their nonlinear graphs were plotted by the capacity of lead adsorbed by adsorbent materials at the time (*q*_t_) versus time (*t*). All linear and nonlinear adsorption kinetics were analyzed by the following Eqs. (10)–(16)^66–69^:

#### Pseudo-first-order kinetic model

10 $${\text{Linear}}:{\text{ln}}\left( {q_{{\text{e}}} - q_{{\text{t}}} } \right) = {\text{ln}}\, q_{{\text{e}}} {-} \, k_{{1}} t$$

11 $${\text{Nonlinear}}:qt = qe(1 - e^{{ - k_{1} t}} )$$

#### Pseudo-second-order kinetic model

12 $${\text{Linear}}:t/q_{{\text{t}}} = {1}/k_{{2}} q_{{\text{e}}}^{{2}} + \, \left( {t/q_{{\text{e}}} } \right)$$

13 $${\text{Nonlinear}}:q_{{\text{t}}} = \, k_{{2}} q_{{\text{e}}}^{{2}} t/\left( {{1} + \, q_{{\text{e}}} k_{{2}} t} \right)$$

#### Elovich model

14 $${\text{Linear}}:q_{t} = { 1}/\beta \, {\text{ln}}\, \alpha \beta + { 1}/\beta\, {\text{ln}} \, t$$

15 $${\text{Nonlinear}}:q_{{\text{t}}} = \beta \, {\text{ln}} \, t + \beta \, {\text{ln}} \, \alpha$$

#### Intra-particle diffusion model

16 $${\text{Linear and nonlinear}}:q_{{\text{t}}} = k_{{\text{i}}} t^{{0.{5}}} + C_{{\text{i}}}$$

where *q*_e_ is the amount of adsorbed lead on adsorbent materials (mg/g), *q*_t_ is the amount of adsorbed lead at the time (mg/g), *k*_1_ is a pseudo-first-order rate constant (min^−1^), and *k*_2_ is a pseudo-second-order rate constant *(*g/mg min). *α* is the initial adsorption rate (mg/g/min) and *β* is the extent of surface coverage *(*g/mg*). k*_i_ is the intra-particle diffusion rate constant *(*mg/g min^0.5^*)* and *C*_i_ is the constant that gives an idea about the thickness of the boundary layer (mg/g).

For adsorption kinetic experiments, 0.175 g of ZB, 0.100 g of ZBF, 0.175 g of ZBB, 0.125 g of ZBFB, and 0.100 g of ZBBF were added to 1000 mL of breaker with the lead concentration of 50 mg/L. The control condition of ZB, ZBF, ZBB, ZBFB, and ZBBF was a sample volume of 1000 mL, a shaking speed of 200 rpm, pH 5, a temperature of 25 °C, and a contact time of 8 h.

## Data Availability

The datasets used and/or analyzed during the current study are available from the corresponding author upon reasonable request.

## References

[1] Balali-Mood M, Naseri K, Tahergorabi Z, Khazdair MR, Sadeghi M (2021). Toxic mechanisms of five heavy metals: Mercury, lead, chromium, cadmium, and arsenic. Front. Pharmacol..

[2] Obeng-Gyasi E (2019). Sources of lead exposure in various countries. Rev. Environ. Health.

[3] Qasem NAA, Mohammed RH, Lawal DU (2021). Removal of heavy metal ions from wastewater: A comprehensive and critical review. NPJ Clean Water.

[4] Ren Y (2021). A mini review of multifunctional ultrafiltration membranes for wastewater decontamination: Additional functions of adsorption and catalytic oxidation. Sci. Total Environ..

[5] Tehreem S (2022). Analysis of the role of various biochar in the remediation of heavy metals in contaminated water and its kinetics study. J. Saudi Chem. Soc..

[6] Pillai P, Kakadiya N, Timaniya Z, Dharaskar S, Sillanpaa M (2020). Removal of arsenic using iron oxide amended with rice husk nanoparticles from aqueous solution. Mater. Today Proc..

[7] Jin X (2022). Functionalized porous nanoscale Fe_3_O_4_ particles supported biochar from peanut shell for Pb(II) ions removal from landscape wastewater. Environ. Sci. Pollut. Res..

[8] Tolkou AK (2022). Chromium(VI) removal from water by lanthanum hybrid modified activated carbon produced from coconut shells. Nanomaterials.

[9] El Nemr A (2021). New magnetic cellulose nanobiocomposites for Cu(II), Cd(II) and Pb(II) ions removal: kinetics, thermodynamics and analytical evaluation. Nanotechnol. Environ. Eng..

[10] Praipipat P, Ngamsurach P, Sanghuayprai A (2023). Modification of sugarcane bagasse with iron(III) oxide-hydroxide to improve its adsorption property for removing lead(II) ions. Sci. Rep..

[11] Ashfaq A (2021). Efficient adsorption of lead ions from synthetic wastewater using agrowaste-based mixed biomass (potato peels and banana peels). Water.

[12] Threepanich A, Praipipat P (2021). Powdered and beaded lemon peels-doped iron(III) oxide-hydroxide materials for lead removal applications: Synthesis, characterizations, and lead adsorption studies. J. Environ. Chem. Eng..

[13] Threepanich A, Praipipat P (2022). Efficacy study of recycling materials by lemon peels as novel lead adsorbents with comparing of material form effects and possibility of continuous flow experiment. Environ. Sci. Pollut. Res..

[14] Wijeyawardana P (2022). Removal of Cu, Pb and Zn from stormwater using an industrially manufactured sawdust and paddy husk derived biochar. Environ. Technol. Innov..

[15] Astuti W, Chafidz A, Al-Fatesh AS, Fakeeha AH (2021). Removal of lead (Pb(II)) and zinc (Zn(II)) from aqueous solution using coal fly ash (CFA) as a dual-sites adsorbent. Chin. J. Chem. Eng..

[16] Suresh S, Sillanpää M, Banat F, Vissa RK (2022). Adsorption of arsenic in aqueous solution onto iron impregnated bagasse fly ash. J. Environ. Health Sci. Eng..

[17] Praipipat P, Ngamsurach P, Kosumphan S, Mokkarat J (2023). Powdered and beaded sawdust materials modified iron (III) oxide-hydroxide for adsorption of lead (II) ion and reactive blue 4 dye. Sci Rep.

[18] Zhang P, Liao W, Kumar A, Zhang Q, Ma H (2020). Characterization of sugarcane bagasse ash as a potential supplementary cementitious material: Comparison with coal combustion fly ash. J. Clean. Prod..

[19] Jangkorn S, Youngme S, Praipipat P (2022). Comparative lead adsorptions in synthetic wastewater by synthesized zeolite A of recycled industrial wastes from sugar factory and power plant. Heliyon.

[20] Shah B, Mistry C, Shah A (2013). Seizure modeling of Pb(II) and Cd(II) from aqueous solution by chemically modified sugarcane bagasse fly ash: Isotherms, kinetics, and column study. Environ. Sci. Pollut. Res..

[21] Oliveira JA, Cunha FA, Ruotolo LAM (2019). Synthesis of zeolite from sugarcane bagasse fly ash and its application as a low-cost adsorbent to remove heavy metals. J. Clean. Prod..

[22] Fungaro DA, Silva KC, Mahmoud AED (2021). Aluminium tertiary industry waste and ashes samples for development of zeolitic material synthesis. J. Appl. Mater. Technol..

[23] Nasrollahzadeh M (2020). Pd nanocatalyst stabilized on amine-modified zeolite: Antibacterial and catalytic activities for environmental pollution remediation in aqueous medium. Sep. Purif. Technol..

[24] Mousavi Khadem SS (2022). MEL zeolite nanosheet membranes for water purification: Insights from molecular dynamics simulations. J. Nanostruct. Chem..

[25] Boycheva S (2020). Studies on the potential of nonmodified and metal of heavy metals and catalytic degradation of organics for waste water recovery. Processes.

[26] Abdellaoui Y (2021). Iron-zirconium microwave-assisted modification of small-pore zeolite W and its alginate composites for enhanced aqueous removal of As(V) ions: Experimental and theoretical studies. Chem. Eng. J..

[27] Peng Z (2021). Removal of cadmium from wastewater by magnetic zeolite synthesized from natural, low-grade molybdenum. Sci. Total Environ..

[28] Neolaka YAB (2022). Efficiency of activated natural zeolite-based magnetic composite (ANZ-Fe_3_O_4_) as a novel adsorbent for removal of Cr(VI) from wastewater. J. Mater. Res. Technol..

[29] Mubarak MF, Mohamed AMG, Keshawy M, elMoghny TA, Shehata N (2022). Adsorption of heavy metals and hardness ions from groundwater onto modified zeolite: Batch and column studies. Alexandria Eng. J..

[30] Alswat AA, Ahmad MB, Saleh TA (2016). Zeolite modified with copper oxide and iron oxide for lead and arsenic adsorption from aqueous solutions. J. Water Supply Res. Technol..

[31] Irannajad M, Haghighi HK (2017). Removal of Co^2+^, Ni^2+^, and Pb^2+^ by manganese oxide-coated zeolite: Equilibrium, thermodynamics, and kinetics studies. Clays Clay Miner..

[32] Dzyazko YS (2022). Hydrated iron oxide embedded to natural zeolite: Effect of nanoparticles and microparticles on sorption properties of composites. Water Air Soil Pollut..

[33] Mays TJ (2007). A new classification of pore sizes. Stud. Surf. Sci. Catal..

[34] Treacy MMJ, Higgins JB (2007). Collection of Simulated XRD Powder Patterns for Zeolites.

[35] Li Y (2022). Controlled fabrication and characterization of α-FeOOH nanorods. J. Inorg. Organomet. Polym. Mater..

[36] Lakouraj MM, Mojerlou F, Zare EN (2014). Nanogel and superparamagnetic nanocomposite based on sodium alginate for sorption of heavy metal ions. Carbohydr. Polym..

[37] Han R (2009). Characterization and properties of iron oxide-coated zeolite as adsorbent for removal of copper(II) from solution in fixed bed column. Chem. Eng. J..

[38] Alkan M, Hopa Ç, Yilmaz Z, Güler H (2005). The effect of alkali concentration and solid/liquid ratio on the hydrothermal synthesis of zeolite NaA from natural kaolinite. Microporous Mesoporous Mater..

[39] Molina A, Poole C (2004). A comparative study using two methods to produce zeolites from fly ash. Miner. Eng..

[40] Scarano D (1993). Fourier-transform infrared and raman spectra of pure and Al-, B-, Ti- and Fe-substituted silicalites: Stretching-mode region. J. Chem. Soc. Faraday Trans..

[41] Xu Y, Axe L (2005). Synthesis and characterization of iron oxide-coated silica and its effect on metal adsorption. J. Colloid Interface Sci..

[42] Li B, Dong Y, Zou C, Xu Y (2014). Iron(III)-alginate fiber complex as a highly effective and stable heterogeneous fenton photocatalyst for mineralization of organic dye. Ind. Eng. Chem. Res..

[43] Ngamsurach P, Praipipat P (2021). Modified alginate beads with ethanol extraction of *Cratoxylum formosum* and *Polygonum odoratum* for antibacterial activities. ACS Omega.

[44] Ngamsurach P, Praipipat P (2022). Antibacterial activities against *Staphylococcus aureus* and *Escherichia coli* of extracted *Piper betle* leaf materials by disc diffusion assay and batch experiments. RSC Adv..

[45] Ngamsurach P, Nemkhuntod S, Chanaphan P, Praipipat P (2022). Modified beaded materials from recycled wastes of bagasse and bagasse fly ash with iron(III) oxide-hydroxide and zinc oxide for the removal of reactive blue 4 dye in aqueous solution. ACS Omega.

[46] Praipipat P, Ngamsurach P, Saekrathok C, Phomtai S (2022). Chicken and duck eggshell beads modified with iron(III) oxide-hydroxide and zinc oxide for reactive blue 4 dye removal. Arab. J. Chem..

[47] Ngamsurach P, Namwongsa N, Praipipat P (2022). Synthesis of powdered and beaded chitosan materials modified with ZnO for removing lead(II) ions. Sci. Rep..

[48] Praipipat P, Ngamsurach P, Prasongdee V (2022). Comparative reactive blue 4 dye removal by lemon peel bead doping with iron(III) oxide-hydroxide and zinc oxide. ACS Omega.

[49] Ngamsurach P, Praipipat P (2022). Comparative antibacterial activities of Garcinia cowa and *Piper sarmentosum* extracts against *Staphylococcus aureus* and *Escherichia coli* with studying on disc diffusion assay, material characterizations, and batch experiments. Heliyon.

[50] Praipipat P, Ngamsurach P, Thanyahan A, Sakda A (2023). Reactive blue 4 adsorption efficiencies on bagasse and bagasse fly ash beads modified with titanium dioxide (TiO_2_), magnesium oxide (MgO), and aluminum oxide (Al_2_O_3_). Ind. Crop. Prod..

[51] Panek R (2021). Simultaneous removal of Pb^2+^ and Zn^2+^ heavy metals using fly ash Na-X zeolite and its carbon Na-X(C) composite. Materials.

[52] Karatas M (2012). Removal of Pb(II) from water by natural zeolitic tuff: Kinetics and thermodynamics. J. Hazard. Mater..

[53] Chen G, Shi L (2017). Removal of Cd(II) and Pb(II) ions from natural water using a low-cost synthetic mineral: Behavior and mechanisms. RSC Adv..

[54] Priyadi IS, Mukti RR (2015). Characteristics of heavy metals adsorption Cu, Pb and Cd using synthetics zeolite Zsm-5. J. Trop. Soils.

[55] Kobayashi Y, Ogata F, Saenjum C, Nakamura T, Kawasaki N (2020). Removal of Pb^2+^ from aqueous solutions using K-type zeolite synthesized from coal fly ash. Water.

[56] Li Z (2018). Zeolite-supported nanoscale zero-valent iron: New findings on simultaneous adsorption of Cd (II), Pb (II), and As (III) in aqueous solution and soil. J. Hazard. Mater..

[57] Rondón W (2013). Application of 3A zeolite prepared from venezuelan kaolin for removal of Pb(II) from wastewater and its determination by flame atomic absorption spectrometry. Am. J. Anal. Chem..

[58] Hussain T (2021). Synthesis and characterization of Na-zeolites from textile waste ash and its application for removal of lead (Pb) from wastewater. Int. J. Environ. Res. Public Health.

[59] Fan X, Liu H, Anang E, Ren D (2021). Effects of electronegativity and hydration energy on the selective adsorption of heavy metal ions by synthetic nax zeolite. Materials.

[60] Rahimi S, Moattari RM, Rajabi L, Derakhshan AA, Keyhani M (2015). Iron oxide/hydroxide (α, γ-FeOOH) nanoparticles as high potential adsorbents for lead removal from polluted aquatic media. J. Ind. Eng. Chem..

[61] Isawi H (2020). Using zeolite/polyvinyl alcohol/sodium alginate nanocomposite beads for removal of some heavy metals from wastewater. Arab. J. Chem..

[62] Langmuir I (1918). The adsorption of gases on plane surfaces of glass, mica and platinum. J. Am. Chem. Soc..

[63] Freundlich H (1906). Over the adsorption in solution. J. Phys. Chem..

[64] Temkin MI, Pyzhev V (1940). Kinetics of ammonia synthesis on promoted iron catalysts. Acta Physiochim. URSS.

[65] Dubinin MM, Radushkevich LV (1947). The equation of the characteristic curve of activated charcoal. Proc. USSR Acad. Sci..

[66] Lagergren S (1898). About the theory of so-called adsorption of soluble substances. K. Sven. Vetensk. Handl..

[67] Ho YS, McKay G (1999). Pseudo-second order model for sorption processes. Process Biochem..

[68] Elovich SY, Larinov OG (1962). Theory of adsorption from solutions of non electrolytes on solid (I) equation adsorption from solutions and the analysis of its simplest form, (II) verification of the equation of adsorption isotherm from solutions. Izv. Akad. Nauk. SSSR Otd. Khim. Nauk.

[69] Weber WJ, Morris JC (1963). Kinetics of adsorption carbon from solution. J. Sanit. Eng. Div..

